# TOX2 nuclear-cytosol translocation is linked to leukemogenesis of acute T-cell leukemia by repressing TIM3 transcription

**DOI:** 10.1038/s41418-024-01352-z

**Published:** 2024-07-30

**Authors:** Anzhou Li, Junbao Zhang, Liangping Zhan, Xiufeng Liu, Xiliang Zeng, Qian Zhu, Zifeng Wang, Jiang Li

**Affiliations:** 1 State Key Laboratory of Oncology in South China, Collaborative Innovation Center for Cancer Medicine, Guangdong Provincial Clinical Research Center for Cancer, Guangdong, China; 2 Department of Biotherapy, Sun Yat-sen University Cancer Center, Guangzhou, China

**Keywords:** Oncogenes, Oncogenes

## Abstract

Nuclear factors TOX and TOX2 upregulate TIM3 expression and lead to T-cell exhaustion in malignancies. Here, we demonstrate two distinct TIM3 expression patterns (high & low) with high TOX and TOX2 levels in T-cell acute lymphoblastic leukemia (T-ALL) specimens and cell lines. However, the mechanisms regulated by TOX and TIM3 signaling in leukemogenesis are unclear. We found that TOX and TOX2 proteins each directly upregulated *HAVCR2* transcription, while the cellular localization of TOX2 was different in Jurkat and MOLT3 cells (nucleus) and lymphoblastic cell T2 and normal T cells (cytoplasm). Nuclear TOX and TOX2 formed a protein complex and repressed *HAVCR2* promoter activity by recruiting transcriptional corepressor LCOR and deacetylase HDAC3. The nuclear-cytosol translocation of TOX2 was deacetylation-dependent and cooperatively mediated by deacetylase Sirt1 and kinase TBK1. Radiation damage induced TOX2 nuclear translocation and decreased Sirt1, TIM3, and caspase 1 expression in normal T cells. Accordingly, knockdown of TOX, TOX2 or LCOR; HDAC3 inhibition; or TIM3 overexpression induced Jurkat cell apoptosis in vitro and slow growth in vivo. Thus, our findings demonstrate a novel regulatory mechanism involving TOX-TOX2 and the TIM3 pathway in the leukemogenesis of T-ALL.

## Introduction

The thymocyte selection-associated high mobility group (HMG) box (TOX) family is composed of TOX (TOX1), TOX2, TOX3, and TOX4 [1]. These TOX members share similar HMG-box DNA-binding domains and genomic organization, as well as an N-terminal domain with transactivation activity and approximately 30–40% sequence identity among the TOX subfamily members [1]. In contrast, the C-terminal domain is specific to different family members, which is suggestive of distinct functions of individual proteins. TOX and TOX2 are highly expressed at specific stages of T-cell development in the thymus [2–5]. TOX has been shown to participate in the regulation of cell apoptosis, growth, metastasis, and DNA repair [6]. Therefore, some researchers think that the TOX family plays an important role in tumorigenesis [6], but the mechanism is still largely unknown. However, TOX has been identified as a crucial factor in tumor-specific T-cell differentiation and CD8+ T-cell exhaustion, linking it to the prognosis of cancers or response prediction, or the enhancement of cancer immunotherapy [7–10].

T-cell immunoglobulin and mucin-domain containing-3 (TIM3), encoded by *HAVCR2*, is a co-inhibitory protein originally found to be expressed on the surface of T helper 1 (Th1) CD4+ T cells and now commonly associated with severely exhausted effector CD8+ T cells [11]. TIM3 has been implicated in driving T-cell apoptosis within the tumor microenvironment [12, 13]. A large amount of experimental data supports TIM3 as an immune checkpoint, and targeting TIM3 is a promising treatment method in current immunotherapy [14–16]. Immune checkpoint molecules can be regulated at multiple levels; for example, in addition to transcriptional regulation, the post-translational modification of PD-1 and PD-L1 has been shown to regulate their protein stability and interaction [17]. TOX and TOX2 have been shown by CHIP-qPCR to upregulate *HAVCR2* transcription [18]. However, few data have detailed the regulation of TIM3 expression in T cells.

We observed that tumor samples from T-cell acute lymphoblastic leukemia (T-ALL) patients and some T-ALL cell lines had high *TOX* and *TOX2* expression together with a low level of TIM3 expression, contradicting previous reports that TOX and TOX2 upregulate TIM3 expression in tumor-specific T cells. Thus, we aim to explore the distinct mechanism of TOX and TOX2 on TIM3 transcription and expression in T-ALL cells, and the role of TOX and TOX2 in mediating TIM3 signaling in the leukemogenesis of T-ALL.

## Results

### Differential expression pattern between TIM3, TOX, and TOX2 in malignancy

**Fig. 1:**
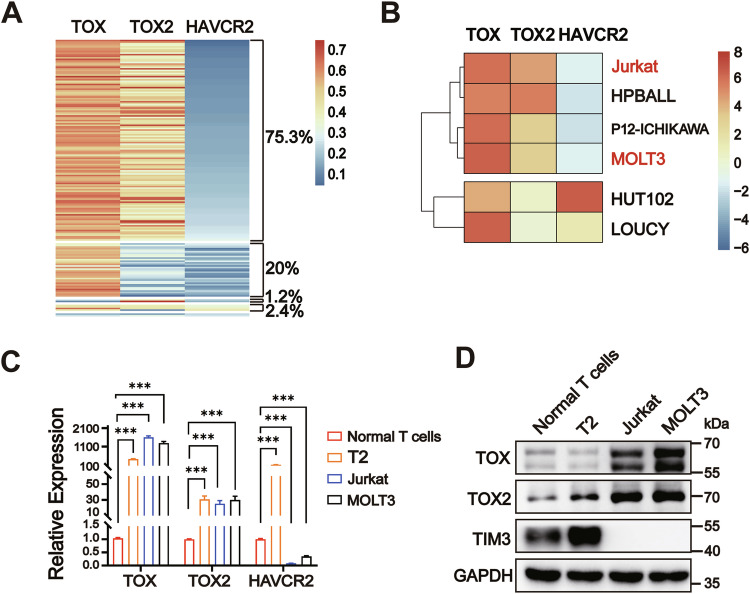
Distinct TIM3 expression pattern with high TOX and TOX2 levels. **A** Heatmap of the mRNA expression of *TOX*, *TOX2*, and *HAVCR2* in bone marrow samples from T-ALL patients (*n* = 170, GSE: 13159). **B** *TOX*, *TOX2*, and *HAVCR2* mRNA expression in human T-ALL cell lines from CCLE database. Expression of *TOX*, *TOX2*, and *HAVCR2* by qPCR (**C**) and immunoblotting (**D**) in OKT3-stimulated normal T cells, T2, Jurkat, and MOLT3 cells. Normal T cells were isolated from healthy PBMCs and activated by OKT3 in vitro for 3 days. Data are mean ± SEM (*n* = 3 biological replicates). ***P < 0.001. Significance was evaluated by one-way ANOVA (**C**). T2: human T-cell lymphoblastic line.

TOX and TOX2 have been reported to induce tumor-specific T-cell exhaustion with upregulation of checkpoint proteins, such as TIM3 and PD-1 [8]. We found a mutual positive association between *TOX* and *TOX2*, *TOX* and *HAVCR2*, and *TOX2* and *HAVCR2* at the transcriptional level in CD3^+^ tumor-infiltrating T cells (TILs) in single cell (sc)RNA-sequencing of nasopharyngeal carcinoma samples (Supplementary Fig. 1). However, most of the patients with T-ALL had low *HAVCR2* mRNA expression with high *TOX* and *TOX2* mRNA levels (75.3%, 128 of 170), and only 24.7% (42/170) of T-ALL patients had high *HAVCR2* mRNA expression with high *TOX* and *TOX2* mRNA levels in bone marrow samples (Fig. 1A). Moreover, the T-ALL cell lines presented two *HAVCR2* expression patterns with high levels of *TOX* and *TOX2*: low *HAVCR2* mRNA expression plus high *TOX* and *TOX2* mRNA levels, such as in Jurkat, HPBALL, P12-ICHIKAWA, and MOLT3 cells, or high *HAVCR2* mRNA expression plus high *TOX* and *TOX2* mRNA levels, such as in HUT102 and LOUCY cells (Fig. 1B). We further determined low TIM3 expression with high TOX and TOX2 levels in Jurkat and MOLT3 cells, and high TIM3 expression with high TOX and TOX2 levels in OKT3-stimulated normal T cells and T-cell lymphoblastic T2 cells using quantitative real-time PCR (qPCR) and immunoblot assays (Fig. 1C, D).

### TOX and TOX2 directly upregulate *HAVCR2* transcription

**Fig. 2:**
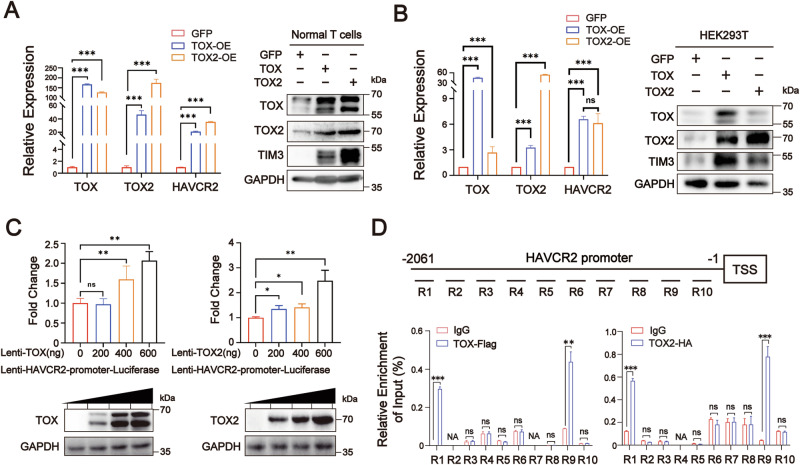
TIM3 transcription is directly regulated by TOX and TOX2. TOX or TOX2 expression was forced in normal T cells and HEK293T cells by transfection with lenti-TOX, TOX2, or GFP-control vector, and T cells were isolated from healthy donors and activated by OKT3 in vitro for 3 days. qRT-PCR analysis (left) and Western blot analysis (right) of the expression of total TOX, TOX2, and TIM3 in T cells (**A**) and HEK293T cells (**B**). **C** Dual luciferase reporter gene assay and immunoblot analysis (bottom) of the *HAVCR2* promoter activity (top) and protein expression in HEK293T cells after transfection with different doses of exogenous TOX- or TOX2-overexpression vector. The lenti-HAVCR2-promoter-luciferase (firefly-luciferase reporter vector) containing *HAVCR2* promoter (2061nt sequence upstream of the transcription start site [TSS]) was co-transfected at a dose of 500 ng with 50 ng renilla-luciferase vector in HEK293T cells for 24 h, and then TOX- or TOX2-overexpression vector was transfected into these HEK293T cells for 48 h at the indicated doses. GAPDH was used as a loading control in **A**–**C**. **D** Chromatin immunoprecipitation-real-time quantitative PCR (ChIP-qPCR) of region 1 – region 10 (R1-R10) in HEK293T cells overexpressing TOX-Flag and TOX2-HA. Immunoprecipitation (IP) was performed using Flag and HA antibodies. R1-R10 indicate the regions predicted to contain 10 similar TOX-binding motifs on the HAVCR2 promoter sequence. Primer sequences are in Supplementary Table 1. Data are means ± SEM (*n* = 3). *P < 0.05, **P < 0.01, ***P < 0.001, unpaired Student’s *t* test (**D**) and one-way ANOVA (**B**). nt nucleotide.

To further address the regulatory mechanism of TIM3 expression in T cells, we forced the expression of TOX or TOX2 in human OKT3-stimulated normal T cells and HEK293T cells. Overexpression of TOX significantly upregulated TOX2 and TIM3 at both the mRNA and protein level, whereas overexpression of TOX2 significantly upregulated TOX and TIM3 at the mRNA and protein level in both T cells and HEK293T cells (Fig. 2A, B). Subsequently, we identified that both TOX and TOX2 significantly enhanced the transcriptional activity of the *HAVCR2* promoter in a dose-dependent manner using dual luciferase assay (Fig. 2C). Homologous alignment revealed that human and mouse TOX proteins had a highly conserved DNA-binding domain HMG-box, and human TOX2 and TOX presented a highly conserved DNA-binding domain HMG-box (Supplementary Fig. 2A, B). Therefore, we analyzed mouse TOX-binding motifs from the mouse ChIP-sequence in a public database (GEO: GSE93953) and predicted 10 TOX DNA-binding motifs in the 2000nt upstream of the human *HAVCR2* promoter among 18 mouse TOX DNA-binding motifs (Supplementary Fig. 2C). ChIP-qPCR analysis using anti-Flag and anti-HA antibodies demonstrated that the ChIP DNA levels of R1 (−2045~−1934nt upstream of the *HAVCR2* promoter) and R9 (−287~−180nt upstream of the *HAVCR2* promoter) were significantly higher in the TOX-Flag and TOX2-HA groups related to IgG control (Fig. 2D). Collectively, these findings provide evidence that both TOX and TOX2 directly upregulate TIM3 expression by binding the *HAVCR2* promoter upstream in the −2045~−1934nt and −287~−180nt regions.

### Nuclear translocation of TOX2 forms a nuclear TOX-TOX2 complex in T-ALL cells

**Fig. 3:**
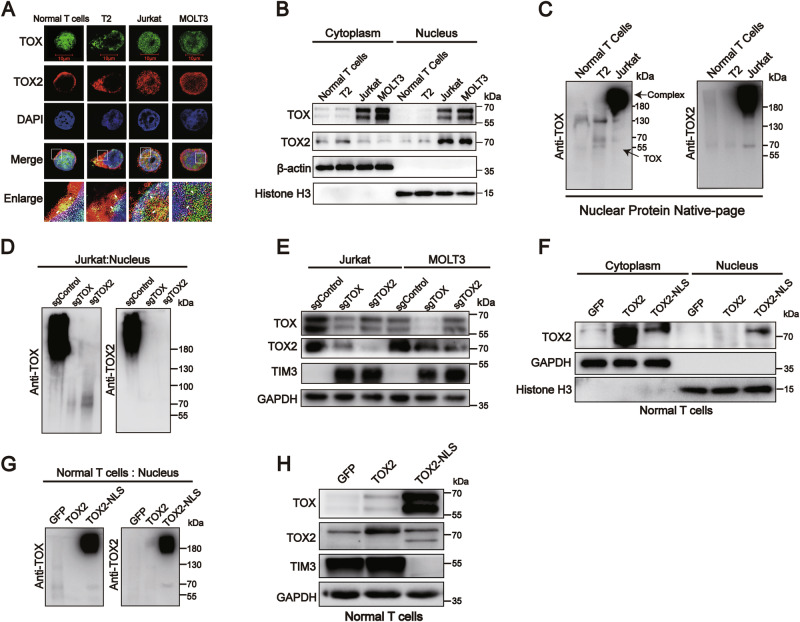
Varying TOX2 cellular localization is associated with TIM3 transcription. **A**–**C** Normal T cells were isolated from healthy PBMCs and activated by OKT3 in vitro for 3 days. **A** Immunofluorescence-laser confocal analysis of TOX (green) and TOX2 (red) cellular localization in OKT3-stimulated normal T cells, T2, Jurkat, and MOLT3 cells. Cell nuclei were stained with DAPI (blue). Yellow: co-localization of TOX and TOX2 as indicated by white arrowheads. **B** Immunoblot analysis detected the cellular distribution of TOX and TOX2 in the cytoplasm and nucleus of OKT3-stimulated normal T cells, T2, Jurkat, and MOLT3 cells. β-actin and histone H3 were used as cytoplasm and nuclear loading controls, respectively. **C** Native-page electrophoresis and immunoblot analysis of the TOX-TOX2 complex at approximately 180 kDa in the nucleus of OKT3-stimulated normal T cells, T2, and Jurkat cells, and TOX dimer at approximately 130 kDa in the nucleus of OKT3-stimulated normal T cells and T2 cells. **D** Jurkat cells transfected with sgTOX, sgTOX2, or control vector were harvested for native-page electrophoresis and immunoblot analysis of the TOX-TOX2 complex in nuclear proteins. **E** Immunoblot analysis detected TIM3 expression in Jurkat and MOLT3 cells transfected with sgTOX, sgTOX2, or control vector. GAPDH was used as a loading control. **F**–**H** Normal T cells were transfected with lenti-GFP (control) or lenti-TOX2/TOX2-NLS in OKT3-coated cell plates for 3 days. **F** Immunoblot analysis of TOX2 protein levels in the cytoplasm and nucleus of TOX2- and TOX2-NLS-T cells. GAPDH and histone H3 were used as cytoplasm and nuclear loading controls, respectively. **G** OKT3-stimulated normal T cells were transfected with lenti-TOX2/TOX2-NLS (nuclear localization sequence) for 72 h and harvested for immunoblot analysis of TIM3 expression. **H** Native-page electrophoresis and immunoblot analysis of the TOX-TOX2 complex in the nucleus of OKT3-stimulated normal T cells overexpressing GFP, TOX2, or TOX2-NLS. Immunoblot analysis was performed as three biological replicates. GAPDH was used as a loading control.

To investigate how TOX and TOX2 regulate the distinct TIM3 expression patterns in T-ALL cell lines, we examined the cellular localization of TOX and TOX2 in OKT3-stimulated normal T cells, T2, Jurkat and MOLT3 cells. TOX was expressed in both the cytoplasm and nucleus in OKT3-stimulated normal T cells, T2, Jurkat, and MOLT3 cells. However, TOX2 was predominantly localized to the nucleus in Jurkat and MOTL3 cells but was mainly expressed in the cytoplasm of T2 and OKT3-stimulated T cells. Thus, TOX and TOX2 co-localized in the nucleus in Jurkat and MOTL3 cells and OKT3-stimulated T cells and the cytoplasm of T2 (Fig. 3A, B). We further demonstrated that, in the nuclei of Jurkat cells, TOX and TOX2 formed a complex (>180 kDa), but in the nuclei of T2 and OKT3-stimulated normal T cells, TOX formed a dimer (~130 kDa) or monomer (55–70 kDa; Fig. 3C). Interestingly, knockdown of TOX or TOX2 decreased the TOX-TOX2 complex in the nucleus and restored the expression of TIM3 in Jurkat and MOLT3 cells (Fig. 3D, E). Surprisingly, overexpression of TOX2 upregulated both TOX and TIM3 expression in normal T cells, but forced TOX2-NLS (exogenous nuclear localization signal) expression upregulated TOX expression and downregulated TIM3 expression. Accordingly, a TOX-TOX2 complex was formed in the nuclear protein of normal T cells with TOX2-NLS overexpression (Fig. 3F–H). These data suggest that the nuclear-cytosol translocation of TOX2 and TOX-TOX2 complex regulates a distinct expression pattern of TIM3 in T-ALL cells.

### Nuclear TOX-TOX2 complex represses *HAVCR2* promoter transcriptional activity

**Fig. 4:**
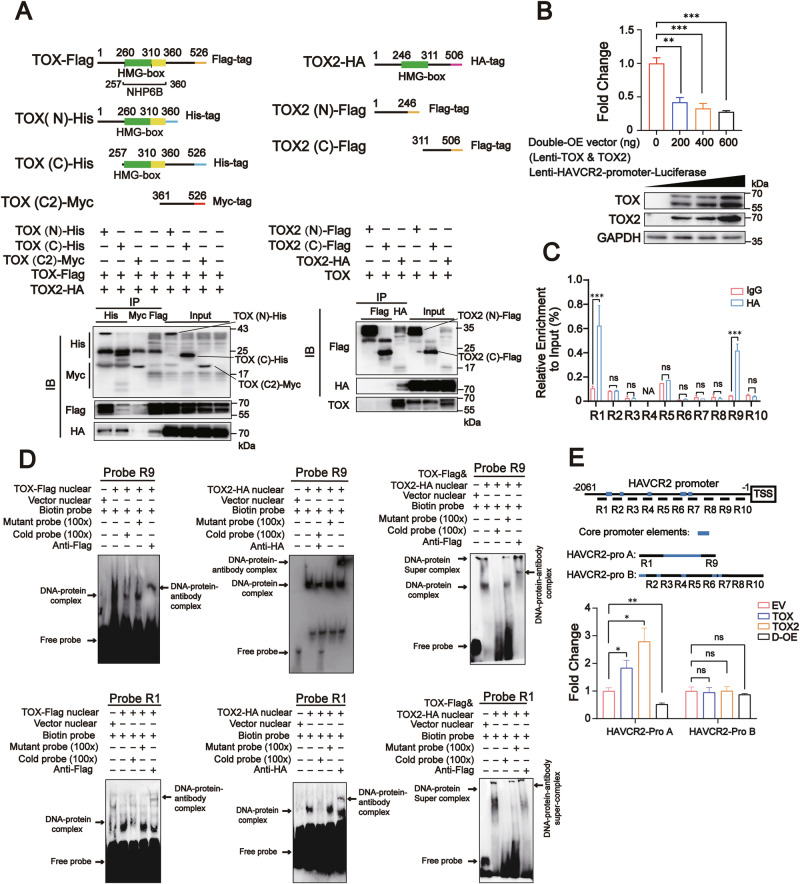
TIM3 transcription was directly suppressed by the TOX-TOX2 complex. **A** Schematic diagrams of the full-length and five truncated TOX or TOX2 plasmids (top). HEK293T cells were transfected with TOX-Flag or TOX2-HA plasmid, and truncated TOX and TOX2 mutant plasmid and coimmunoprecipitation assay performed to detect the interaction between TOX and TOX, or TOX and TOX2 (bottom). **B** Dual luciferase reporter gene assay and immunoblot analysis of the TIM3 transcriptional activity (top) and TOX and TOX2 protein levels (bottom) in HEK293T cells con-transfected with TOX and TOX2 vector at the indicated doses. The lenti-HAVCR2-promoter-luciferase containing HAVCR2 promoter (2061 bp sequence upstream of TSS) was transfected to HEK293T cells. Equal amounts of TOX and TOX2 vectors were co-transfected into HEK293T cells at different doses for 48 h. GAPDH was used as a loading control. **C** The ChIPed DNAs were examined by qPCR for the indicated region of the HAVCR2 promoter. HEK293T cells were co-transfected with equal amounts of TOX-Flag and TOX2-HA for 48 h, and harvested for ChIP analysis using IgG or HA antibody. **D** EMSA of the nuclear protein extracted from HEK293T cells transfected with the vectors containing TOX-Flag, TOX2-HA, or both and bound to biotin-labeled DNA probe R1 (bottom) or R9 (top) from the DNA R1 or R9 region of the HAVCR2 promoter. Arrows indicate the free probe, DNA-protein shift band, and DNA-protein-antibody supershift band. **E** Dual luciferase reporter gene assay of the truncated HAVCR2 promoter A (HAVCR2-pro A: spliced together by −2061~−1694, −1612~−1563, −1033~−984, −679~−630, −575~−525 and −227~−180nt region of HAVCR2 promoter upstream from TSS) and HAVCR2 promoter B (HAVCR2-pro B: spliced together by −1831~−227, −180~−1nt region of HAVCR2 promoter upstream from TSS) in HEK293T cells transfected with TOX-Flag, TOX2-HA, both, or control vector. Renilla-luciferase vector (50 ng) was co-expressed with 500 ng of HAVCR2-pro A and HAVCR2-pro B in HEK293T cells and 600 ng of TOX and TOX2 individually or together in HEK293T for 48 h. Data are means ± SEM. *P < 0.05, **P < 0.01, ***P < 0.001, ns not significant, unpaired Student’s *t* test (**C**) and one-way ANOVA (**B** and **E**). Images are representative of three independent experiments.

To explore the binding of TOX and TOX2 and TOX-TOX dimer, we generated five truncated TOX or TOX2 plasmids, including TOX(N)-His (1–360), TOX(C)-His (257–526), TOX(C2)-Myc (361–526), TOX2(N)-Flag (1–246), and TOX2(C)-Flag (311–506), and transfected them into HEK293T cells with full-length TOX, TOX-Flag, or TOX2-HA plasmids (Fig. 4A). TOX could bind with TOX(N)-His, TOX(C)-His, and TOX2-HA, but not TOX(C2)-Myc, TOX2(N)-Flag, or TOX2(C)-Flag. TOX2-HA could bind with TOX(N)-His, TOX(C)-His, and full-length TOX, but not TOX(C2)-Myc in exogenous Co-IP analysis (Fig. 4A). These data suggest that TOX and TOX or TOX and TOX2 bind at 257–360aa, the Nuclear Histone Paralogous Protein 6B (NHP6B) domain containing the HMG-box domain (260–310aa) in TOX and the HMG-box domain (246–311aa) in TOX2. We further determined that TOX-TOX2 complex (>180 kDa) was present in HEK293T cells with forced TOX and TOX2 expression; a 130 kDa TOX dimer was found in HEK293T cells with TOX overexpression and TOX2 knockdown, and a 55–70 kDa TOX2 monomer in HEK293T cells with TOX knockdown and TOX2 overexpression (Supplementary Fig. 3B). We also observed that *HAVCR2* promoter transcriptional activity was suppressed by the co-expression of TOX-Flag and TOX2-HA in a dose-dependent manner in dual-luciferase reporter gene assays (Fig. 4B and Supplementary Fig. 3C). To further determine the location of TOX-TOX2 complex binding in the *HAVCR2* promoter, we detected the ChIP enrichment signals at the R1 to R10 sites of the *HAVCR2* promoter using anti-HA ChIP-qPCR in HEK293T cells co-transfected with TOX-Flag and TOX2-HA and determined that the anti-HA group could specifically pull-down R1 (−2045~−1934nt upstream of the *HAVCR2* promoter) and R9 (−287~−180nt upstream of the *HAVCR2* promoter) DNA sequences (Fig. 4C). We further labeled the DNA sequence in the R1 (TOX motif1) and R9 (TOX motif9) regions as a biotin-labeled probe and demonstrated that the individual TOX and TOX2 proteins and the TOX-TOX2 protein complex could bind to the probes motif1 and motif9 in electrophoretic mobility shift assays (EMSAs) (Fig. 4D). We also generated truncated *HAVCR2* promoters, including HAVCR2-pro A (R1 and R9 nucleotide region) and HAVCR2-pro B (R2 to R8 and R10 nucleotide), and found that TOX and TOX2 induced HAVCR2-pro A transcriptional activity but the TOX-TOX2 complex suppressed HAVCR2-pro A transcriptional activity. However, TOX, TOX2, or the TOX-TOX2 complex had no effect on the HAVCR2-pro B transcriptional activity (Fig. 4E).

### TOX-TOX2 complex represses *HAVCR2* transcription by recruiting co-repressor LCOR

**Fig. 5:**
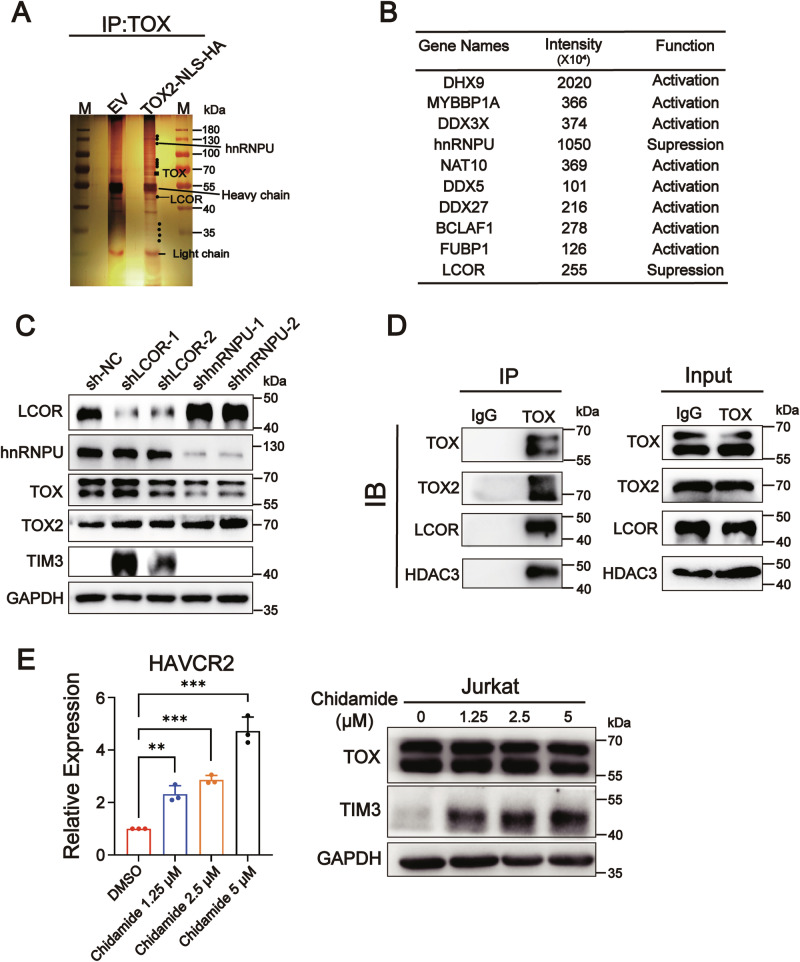
The TOX-TOX2 complex recruits inhibitor LCOR to suppress TIM3 expression. **A**, **B** Normal T cells were isolated from healthy donors and transfected with lentivirus containing empty vector (EV) or TOX2-NLS-HA vector in OKT3-coated plates for 3 days. The two groups of nuclear proteins were immunoprecipitated by anti-TOX and subjected to SDS-PAGE and silver staining (**A**) or mass spectrometry (**B**). M, protein marker; the black dots represent proteins enriched in the TOX2-NLS-HA sample but absent from the EV sample. **C** Immunoblot analysis of protein expression of LCOR, hnRNPU, TOX, TOX2, and TIM3 in Jurkat cells in which LCOR and hnRNPU were knocked down by shLCOR-1, shLCOR-2, shhnRNPU-1, and shhnRNPU-2, respectively. GAPDH was used as a loading control. **D** Co-IP analysis of TOX2, LCOR, and HDAC3 interacting with TOX in the nucleus of Jurkat cells. Immunoprecipitation was performed using anti-TOX antibody. **E** qRT-PCR and immunoblot analysis of the mRNA and protein expression of TIM3 in Jurkat cells after the administration of chidamide at different doses for 24 h. GAPDH was used as a loading control. Data are means ± SEM. **P < 0.01, ***P < 0.001, ns not significant, unpaired Student’s *t* test (**C**) and one-way ANOVA (**E**). Images are representative of three independent experiments.

Based on the above results that the TOX-TOX2 complex, TOX, and TOX2 share a common binding region in the *HAVCR2* promoter (Figs. 2D and 4C–E), we expected that the repression of *HAVCR2* transcription by the TOX-TOX2 complex may be due to the recruitment of transcription suppressors. In nuclear protein Co-IP-silver staining analysis, we found additional protein bands in TOX2-NLS-HA vector-transfected T cells related to control vector-transfected T cells (Fig. 5A). We identified eight transcriptional activators and two transcriptional suppressors, LCOR and hnRNPU, which have been reported to repress gene transcription [19, 20], in IP products from TOX-NLS-HA-transfected T cells in mass spectrum (MS) analysis (Fig. 5B, Supplementary Fig. 3D, and Supplementary File 2). We further determined that shLCOR-1 and -2, but not shhnRNPU-1 and -2, restored the TIM3 protein expression in Jurkat cells (Fig. 5C). LCOR exhibits both deacetylase HDAC-dependent and -independent transcriptional repression [21]. We predicted in silico that LCOR potentially interacts with the deacetylase HDAC3 using the Biogrid database (https://thebiogrid.org/) (Supplementary Fig. 3E). TOX2, HDAC3, and LCOR were bound with TOX in the nuclei of Jurkat cells in Co-IP analysis (Fig. 5D). Furthermore, we demonstrated that TIM3 mRNA and protein expression were restored in Jurkat cells in the presence of chidamide, an inhibitor of HDAC3, in a dose-dependent manner (Fig. 5E).

### Nuclear translocation of TOX2 is deacetylate-dependent and mediated by deacetylase Sirt1 in cooperation with kinase TBK1

**Fig. 6:**
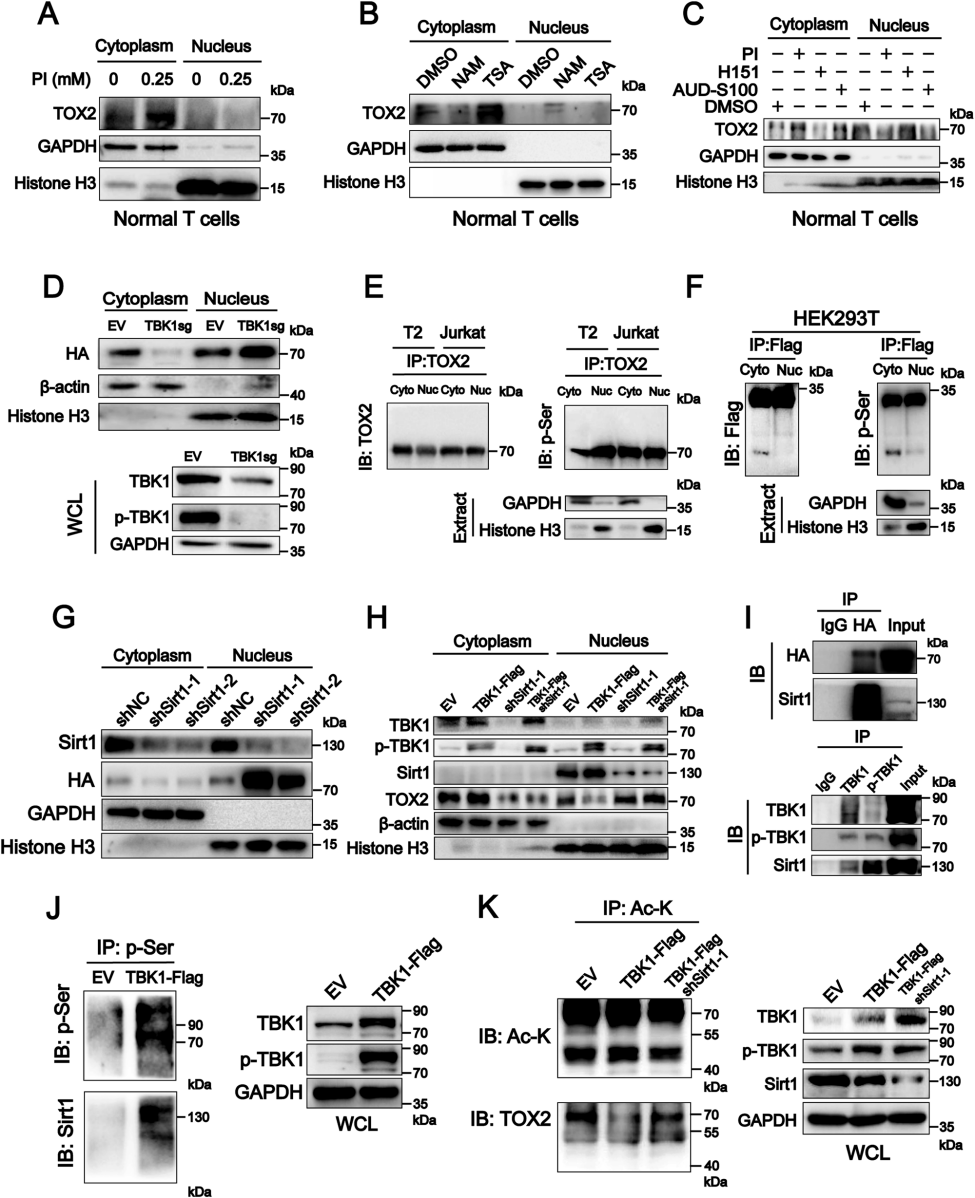
TOX2 nuclear translocation was acetylation-dependent and mediated by deacetylase Sirt1 and phosphokinase TBK1. Immunoblot analysis of the TOX2 levels in the cytoplasm and nucleus in OKT3-stimulated normal T cells after treatment with phosphatase inhibitor (PI) (**A**) or deacetylase inhibitor TSA and NAM (**B**), or phospho-TBK1 (p-TBK1) inhibitor H-151 (10 µM), PI (0.25 mM), and TBK1 activator AUD-S100 (7 µM) (**C**) for 24 h at the indicated concentrations. β-actin and GAPDH were the cytoplasm loading controls, and histone H3 was the nuclear loading control. **D** Immunoblot analysis of TOX2-HA in the cytoplasm and nucleus using anti-HA after the TBK1-sgRNA plasmid and empty-vector (EV) were transfected into TOX2-HA-HEK293T for 48 h. GAPDH was used as a loading control. **E** Immunoprecipitation and immunoblot analysis of endogenous TOX2 and phospho-TOX2 in the cytoplasm and nucleus of T2 and Jurkat cells. IP: anti-TOX2; IB: anti-p-Ser. **F** Immunoprecipitation and immunoblot analysis show exogenous phospho-TOX2(N) and phospho-TOX2(C) levels in the cytoplasm and nucleus of HEK293T cells overexpressing TOX2(N)-TOX2(C)-Flag. IP: anti-Flag; IB: anti-p-Ser. **G** Immunoblot analysis of Sirt1 and TOX2-HA in the nucleus and cytoplasm of TOX2-HA-HEK293T cells transfected with EV, shSirt1-1, and shSirt1-2. **H** Immunoblot analysis of TBK1, p-TBK1, Sirt1, and TOX2 in the nucleus and cytoplasm of TBK1-Flag-, shSirt1-1, and TBK1-Flag/shSirt1-1-Jurkat cells. GAPDH was used as a cytoplasm loading control, and histone H3 was used as a nuclear loading control in **E**–**H**. **I** Co-IP analysis of the nuclear protein extract from HEK293T cells overexpressing TOX2-HA and TBK1. IP: anti-HA, anti-TBK1, or anti-p-TBK1 as indicated; IB: anti-Sirt. **J** Immunoprecipitation and immunoblot analysis of phospho-Sirt1 (p-Ser) in the nucleus of Jurkat cells overexpressing TBK1-Flag. IP: anti-p-Ser; IB: anti-Sirt1. **K** Immunoprecipitation and immunoblot analysis of acetylated TOX2 in TBK1-Flag- and TBK1-Flag/shSirt1-Jurkat cells. IP: anti-acetylated (Ac)-lysine (Ac-K). Whole cell immunoblotting of TBK1, p-TBK1, and Sirt1 in TBK1-Flag- and TBK1-Flag/shSirt1-Jurkat cells as indicated (right). **J**, **K** GAPDH was used as a loading control. Images are representative of three independent experiments. IP Immunoprecipitation, IB Immunoblotting.

Phosphorylation and acetylation have been reported as the two main protein modifications linked to protein nuclear translocation [22–28]. The numbers of serine phosphorylation sites and lysine acetylation sites were identified in the protein sequence of TOX2 in a bioinformatic analysis (Supplementary Fig. 4A, B). Thus, we treated OKT3-stimulated normal T cells with phosphatase inhibitor (PI) and deacetylase inhibitors. The TOX2 expression level was decreased in the nucleus but increased in the cytoplasm of PI-treated OKT3-stimulated normal T cells (Fig. 6A). In contrast, the TOX2 level was increased in the nucleus but decreased in the cytoplasm of OKT3-stimulated normal T cells treated with class III deacetylase Sirt1 inhibitor NAM, but not TSA (Fig. 6B). Subsequently, we predicted in silico using the STRING database (https://cn.string-db.org/) that TBK1 directly binds deacetylase Sirt1 and the TOX2-AKT3 complex binds Sirt1 (Supplementary Fig. 4C). We demonstrated that the increase in p-TBK1 occurred alongside an increase in TOX2 in the cytoplasm of T cells but a decrease in TOX2 in the nuclei of T cells treated with PI and ADU-S100 (STING activator); a decrease in p-TBK1 occurred alongside a decrease in TOX2 in the cytoplasm of T cells but an increase in TOX2 in the nuclei of T cells treated with H151 (p-TBK1 inhibitor). A similar nuclear translocation of TOX2 was increased in HEK293T cells with TBK1 knockdown (Fig. 6C, D). These data suggest that TBK1 inhibits TOX2 nuclear translocation. Interestingly, the TBK1 level was lower in Jurkat cells than T2 and T cells (Supplementary Fig. 4D). However, the level and molecular weight of the serine phosphorylation of TOX2 was similar in the nucleus and cytoplasm of T2 and Jurkat cells in TOX2-IP protein analysis (Fig. 6E). Furthermore, the level and molecular weight of the serine phosphorylation of TOX2(N)-Flag were similar in the cytoplasm and nucleus of HEK293T cells co-transfected with exogenous TOX2(N)-Flag and TOX2(C)-Flag, but the serine phosphorylation of TOX2(C)-Flag was only observed in the cytoplasm (Fig. 6F). These data suggest that TOX2 nuclear translocation is independent of TOX2 protein phosphorylation, and that the effect of TBK1 on the nuclear translocation of TOX2 is indirect. We further examined whether deacetylase Sirt1 is involved in the nuclear translocation of TOX2 and found that the TOX2 level was increased in the nuclei of shSirt1-HEK293T cells (Fig. 6G). Quantitative RT-PCR analysis showed that Sirt1 was expressed at higher levels in Jurkat cells related to T2 and normal T cells (Supplementary Fig. 4E). We proposed that TBK1 may decrease the TOX2 nuclear translocation mediated by Sirt1 based on the above results (Supplementary Fig. 4C). We observed that NAM reversed the decrease in TOX2 nuclear translocation in Jurkat cells overexpressing TBK1, along with the changes in TIM3 expression, and knockdown of Sirt1 showed a similar recovery of TOX2 nuclear translocation in TBK1-Flag-Jurkat cells (Fig. 6H, Supplementary Fig. 4F). In addition, TOX2 could bind Sirt1, and TBK1 and p-TBK1 could bind with Sirt1 in the nuclei of HEK293T cells overexpressing both TBK1 and TOX2 (Fig. 6I). Furthermore, TBK1 overexpression increased the phosphorylation of Sirt1 in the nuclei of Jurkat cells (Fig. 6J). We demonstrated that overexpression of TBK1 decreased the acetylation of TOX2 in Jurkat cells, whereas shSirt1-1 reversed the decreased acetylation of TOX2 mediated by TBK1 (Fig. 6K).

### Radiation damage induces T-cell anti-apoptosis by Sirt1-mediated TOX2 nuclear translocation

**Fig. 7:**
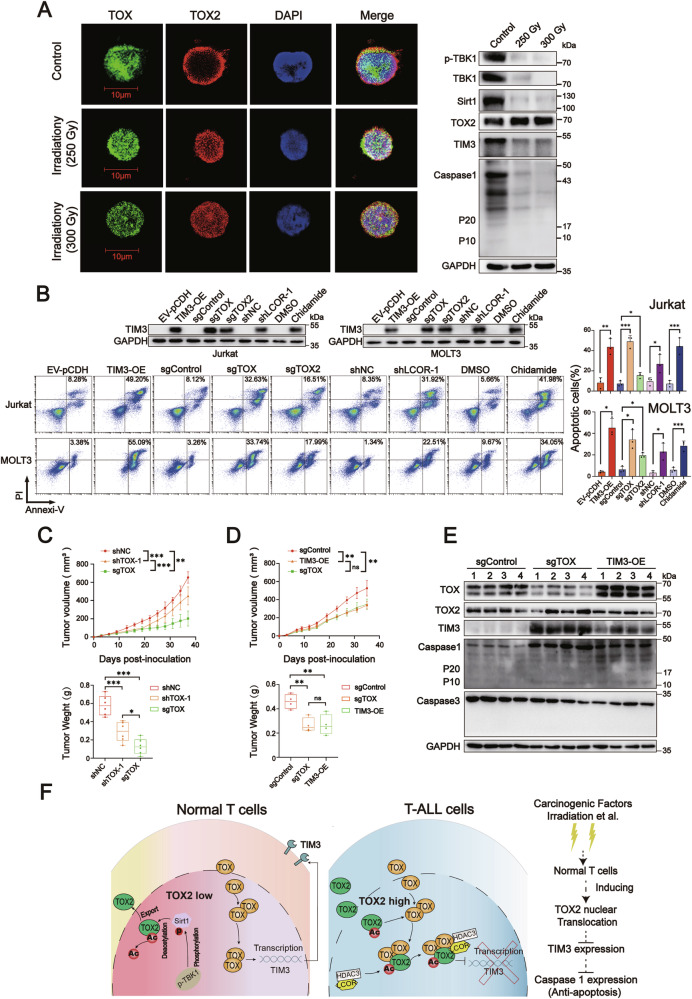
Irradiation damage induces TIM3-mediated anti-apoptosis in normal T cells but TOX-TOX2 complex inhibition induces Jurkat cell apoptosis in vitro and in vivo. **A** Immunofluorescence indicating TOX2 cellular localization in X-ray-irradiated or normal T cells (left) and whole cell immunoblot analysis (right) of the expression of TIM3 and Sirt1 at the indicated dose of X-ray-irradiated T cells and normal T cells. GAPDH was used as a loading control. **B** Representative immunoblot (upper) and flow cytometry (bottom left) with the statistical graph (bottom right) showing TIM3 expression and percentage of apoptotic Jurkat and MOLT3 cells under the indicated treatment conditions. The Jurkat and MOLT3 cells were treated as indicated and cultured for 5 days before harvesting for immunoblotting and flow cytometry. **C** The growth curve and tumor weight of subcutaneous tumors in shNC-, shTOX-1-, and sgTOX-Jurkat xenograft groups (*n* = 6 per group). **D** The growth curve and tumor weight of subcutaneous tumors in sgControl-, TIM3-OE-, and sgTOX-Jurkat xenograft groups (*n* = 4 per group). **E** Immunoblot of the expression of TOX, TOX2, TIM3, caspase 1, and caspase 3 in tumor tissues from sgControl-, sgTOX-, and TIM3-OE-Jurkat tumors. GAPDH was used as a loading control. **F** Schematic of the regulatory mechanism of TIM3 mediated by TOX-TOX2 in normal T cells and Jurkat cells, as well as TOX2 nuclear translocation and activation of anti-apoptosis signaling in normal T cells induced by carcinogenic factors, such as irradiation damage. Data are means ± SEM. *P < 0.05, **P < 0.01, ***P < 0.001, unpaired Student’s *t* test and one-way ANOVA. Data are from three independent experiments.

We observed that X-ray-irradiated OKT3-stimulated normal T cells had increased TOX2 nuclear translocation and decreased P-TBK1, TBK, Sirt1, TIM3, and caspase 1 levels, suggesting that radiation damage, which is a carcinogenic factor, could induce TBK1/Sirt1-mediated TOX2 nuclear translocation and TIM3-mediated anti-apoptosis in normal T cells (Fig. 7A). To further confirm the role of TIM3 and TOX2 nuclear translocation in T-ALL cells, we forced TIM3 expression, knocked out TOX or TOX2 expression, or inhibited TOX2 nuclear translocation using shLCOR-1 or HDAC3 inhibitor chidamide in Jurkat cells. Apoptosis and the TIM3 level were increased in Jurkat cells with administration of sgTOX, sgTOX2, TIM3-OE, and shLCOR-1 vector or chidamide in vitro (Fig. 7B). Importantly, Jurkat-TOXsg-tumor, Jurakat-shTOX-tumor and Jurkat-TIM3-tumor exhibited slower growth than Jurkat-sgControl-tumor in nude mice (Fig. 7C, D). In addition, TIM3 and caspase 1 expression were increased in tumor samples from Jurkat-TOXsg-tumor and Jurkat-TIM3-tumor related to Jurkat-sgControl -tumor, but caspase 3 levels were not changed in these three tumor tissues (Fig. 7E and Supplementary Fig. 5A–C). Taken together, these in vitro and in vivo data suggest that TOX2 nuclear translocation is involved in the leukemogenesis of T-ALL by preventing TIM3-mediated apoptosis of T-ALL cells.

## Discussion

In chronic viral infection or tumors, TOX and TOX2 upregulation is linked to T-cell exhaustion by increasing the levels of suppressive molecules, such as PD-1 and TIM3 [7, 8, 18, 28]. Though TIM3 is usually increased in both the peripheral blood and bone marrow of patients with T-ALL, tumor TIM3 expression is a favorable predictor in T-ALL patients [29, 30]. However, the role of TOX and TOX2 in hematological malignancy originating from T cells and its regulatory mechanism are largely unclear. Here, we observed two distinct TIM3 expression patterns (high & low) in T-ALL cell lines or tumor samples with high *TOX* and *TOX2* levels based on a GEO public database analysis. Our data demonstrate that the individual TOX or TOX2 proteins can directly induce TIM3 transcription and expression. However, we determined that the distinct TIM3 expression in T-ALL cell line Jurkat and MOLT3, lymphoblastic cell line T2, and OKT3-stimulated normal T cells was induced by the nuclear-cytosol translocation of TOX2, and nuclear TOX and TOX2 formed a complex that suppressed *HAVCR2* transcription by recruiting co-suppressor LCOR and acetylase HDAC3 (Sirt1). We further demonstrated that TBK1 and Sirt1 regulate the nuclear translocation of TOX2 by changing the level of acetylated TOX2, resulting in less nuclear export of TOX2 protein. In addition, radiation damage, a common carcinogenic factor for T-ALL, could induce the nuclear translocation of TOX2 in OKT3-stimulated normal T cells, leading to TIM3-mediated cell anti-apoptosis. Targeting TOX or TIM3 overexpression slowed Jurkat-tumor growth in nude mice. Thus, the TOX/TOX2-TIM3 axis plays an important role in the leukemogenesis of malignancies of T cell origin (Fig. 7F).

TOX and TOX2 proteins function as enhancers of “exhausted-related” *PDCD1* transcription [18]. In this study, we found that, individually, TOX and TOX2 could directly upregulate *HAVCR2* transcription as enhancers by binding the −2045~−1934nt and −287~−180nt regions in the *HAVCR2* promoter upstream from the transcription start site (TSS). Explaining the different TIM3 expression patterns in normal T cells (high TIM3 & high TOX and TOX2) and some T-ALL cells (low TIM3 & high TOX and TOX2), we found that a different TOX2 cellular location was linked to different TIM3 expression and nuclear TOX2 binding with TOX, resulting in suppression of *HAVCR2* transcriptional activity in T-ALL cells, such as Jurkat and MOLT3 cells. We further identified the binding site of the TOX-TOX2 complex at TOX (260–360aa) and TOX2 (246–311aa), which is the HMG-box domain of TOX and TOX2. The HMG-box of TOX and TOX2 has also been reported to be the DNA-binding domain [2]. Here, we further uncovered that the TOX-TOX2 complex repressed *HAVCR2* transcription by binding the R1 (−2045~−1934nt) and R9 (−287~−180nt) regions of the *HAVCR2* promoter, the same binding sequence as individual TOX and TOX2 proteins. In addition, we discovered that the TOX-TOX2 complex recruited ligand-dependent co-repressor LCOR and RNA-binding protein hnRNPU in the nuclear protein anti-TOX Co-IP enrichment with silver staining and mass spectrometry analysis, and both proteins have been reported to function in transcriptional repression [19–21]. We further determined that LCOR is involved in TOX-TOX2 complex-mediated repression of *HAVCR2* transcription, and knockdown LCOR could recover the expression of TIM3. This result suggests that the TOX-TOX2 complex inhibits *HAVCR2* transcription through LCOR recruitment. LCOR acts as a transcriptional repressor in two manners: HDAC-dependent or HDAC-independent [21]. Network analysis based on the biogrid database found that LCOR potentially interacts with the deacetylase HDAC3. We found a TOX2, LCOR, and HDAC3 protein complex in the anti-TOX Co-IP enrichment pulling down nuclear protein from Jurkat cells and the HDAC3 inhibitor chidamide restored TIM3 expression in Jurkat cells in a dose-dependent manner. These results suggest that nuclear TOX2 binds with TOX and forms a TOX-TOX2 complex to recruit the transcription inhibitor LCOR and deacetylase HDAC3 in the nucleus, and this protein compound inhibits the transcriptional activity of the *HAVCR2* promoter.

Acetylation and phosphorylation are major manners of regulating protein nuclear-cytosol translocation [23, 25, 26]. Here, we predicted multiple serine phosphorylation sites in the TOX2 protein and lysine acetylation sites at K303 and K380 in the NLS domain of TOX2, and the protein kinase TBK1, ATK3, and deacetylase Sirt1 interacted with TOX2 based on network analysis (Supplementary Fig. 4C). We also found that PI and NAM decreased the nuclear TOX2 levels in normal T cells. Interestingly, TBK1 and p-TBK1 expression was very low in Jurkat cells (TOX2 in nucleus) related to T2 and normal T cells (TOX2 in cytoplasm) (Supplementary Fig. 4D). This inferred that TBK1 may be involved in the regulation of TOX2 nuclear translocation. We further determined that TBK1 was involved in regulation of the nucleus-cytosol translocation of TOX2 through blockade of TBK1 or p-TBK1 in normal T cells. However, the weight of p-serine in anti-TOX2 IP enrichment was similar in the cytoplasm and nucleus in T2, Jurkat, or HEK293T-TOX2-overepxressing cells. These data indicate that the phosphorylation of TOX2 itself does not mediate the nucleus-cytosol translocation of TOX2.

Subsequently, we investigated whether acetylation directly regulates the nuclear-cytosol translocation of TOX2. We first found out that NAM and shSirt1 enhanced nuclear TOX2 expression in normal T cells. TIM3 expression and nuclear TOX2 levels were increased by overexpression of TBK1 but reversed by Sirt1 knockdown, and these data suggest that TBK1 regulates TOX2 nuclear translocation through Sirt1 expression. Deacetylase Sirt1 is a nuclear protein and executes deacetylation modifications in the cell nucleus, and phosphorylation of Sirt1 is the activated status [31–33]. Importantly, we found a compound of TOX2, TOX, Sirt1, TBK1, and p-TBK1 in the nuclei of Jurkat cells and an increase in phosphorylated Sirt1, with a decrease in acetylated TOX2, in the nuclei when TBK1 was overexpressed. Some researchers have found that Sirt1 upregulation could promote the stemness, metastasis, and radio-resistance of tumor cells by mediating protein deacetylation, and leading to protein nuclear-cytoplasmic translocation [34, 35].

As we know, TIM3 is a biomarker of exhausted T cells and induces T cells to undergo apoptosis and death [12, 36]. In T-ALL patients, TIM3 is upregulated in the peripheral blood and linked to poor outcomes [29, 37], but we found that most T-ALL patients had low *HAVCR2* expression in tumor tissues. In addition, TOX2 has been identified as a oncogene and linked to the worse patient survival in natural killer/T-cell lymphoma (NKTL) [38]. Radiation damage is a common cause of pathogenesis in T-ALL [39, 40]. Here, we observed that high doses of irradiation induced the nuclear translocation of endogenous TOX2 and suppressed TIM3 and caspase 1 expression in normal T cells. Accordingly, knockdown of TOX, TOX2, or LCOR, overexpression of TIM3, or inhibition HDAC3 expression induced apoptosis and increased TIM3 levels in Jurkat and MOLT3 cells in vitro. Importantly, Jurkat cells with TOX knockdown or TIM3 overexpression slowed the tumor growth in nude mice. Considering that the TIM-3 function is linked to T-cell exhaustion and dysfunction in the anti-tumor immunity of cancer patients and TIM3 is usually increased in both the peripheral blood and bone marrow of patients with T-ALL [29, 41, 42], we think that the mechanism and effect of targeting TOX/TOX2-TIM3 axis in anti-T-ALL therapy will be complex and limited.

In summary, our study provides further insight into the mechanism of *HAVCR2* transcription mediation by TOX, TOX2, and TOX-TOX2 complex. Individually, TOX and TOX2 can directly induce *HAVCR2* transcription in T cells and T-ALL cells, and the nuclear TOX-TOX2 complex represses *HAVCR2* transcription by recruiting transcription suppressor LCOR and acetylase HDAC3 in T-ALL cells. Deacetylase Sirt1 is phosphorylated by TBK1, and p-Sirt1 functions in the deacetylation of acetylated TOX2, leading to TOX2 nuclear export and subsequent decrease in the *HAVCR2* transcription repression mediated by the TOX-TOX2 complex in normal T cells. Importantly, carcinogenic factors, such as radiation damage, could induce TOX2 nuclear translocation and formation of the TOX-TOX2 complex and suppress TIM3-induced apoptosis signaling in normal T cells. Thus, our findings provide a novel regulatory mechanism involving TOX-TOX2 and the TIM3 pathway in the leukemogenesis of T-ALL (Fig. 7F).

## Materials and methods

### Cell lines and nude mouse tumor model

HEK293T, T2, Jurkat, MOLT3 cells were maintained in our laboratory and cultured in DMEM or RPMI 1640 medium supplemented with 10% fetal bovine serum (ExcellBio, China). Peripheral blood mononuclear cells (PBMCs) were isolated from healthy individuals and then frozen for further analysis. Briefly, PBMCs were plated in 48- or 24-well plates with OKT-3 (1 µg/ml) in low-dose rhIL-2 (150 IU/ml) medium for different treatment conditions. Research involving human subjects, human material, or human data was conducted in accordance with the Declaration of Helsinki. All patients and healthy donors provided written consent. The study was approved by the Research Ethics Committee of Sun Yat-sen University Cancer Center (GZR2022-117). For the nude mice tumor model, before the experiment begins, divide the mice into three experimental groups based on their body weight, ensuring that the average weight of the mice in each group does not exceed 1 g. During the experiment, if the weight loss of the mouse reaches more than 20%, the mouse will be excluded from the experiment and euthanized humanely. 5-week-old female Balb/C nude mice (4 per group or 6 per group) were injected subcutaneously under the left rib with 5 × 10^6^ Jurkat-sgControl/sgTOX/TIM3-OE cells and 10^7^ Jurkat-shNC/shTOX-1/sgTOX cells resuspended in matrix gel (Beyotime, China) diluted with PBS in a 1:1 ratio [43]. The tumor volume was measured every 3 days starting on the third day, and the tumor tissue was finally dissected and tumor weight measured. None blinding and randomization was used. Mice were maintained in the Animal Facility of the Sun Yat-sen University Cancer Center under specific pathogen-free conditions. All mouse experiments were performed according to the regulations of Sun Yat-sen University (L025503202311016).

### Plasmid construction, lentivirus production, and transduction

Full-length and truncated TOX and TOX2 and full-length TBK1 and TIM3 were subcloned into pCDH vector with a C-terminal Flag/His/Myc/HA-epitope tag and 5’XbaI and 3’ XhoI endonuclease sequences. TOX2-NLS plasmid was full-length TOX2 fragments subcloned into the pCDH vector with a C-terminal exogenous NLS (Supplementary Table 1), and the TOX2-NLS-HA plasmid was full-length TOX2 fragments subcloned into the pCDH vector with C-terminal exogenous NLS-HA-epitope sequences. The pro-TIM3 dual-luciferase plasmid contained 2061 bp upstream of the *HAVCR2* promoter ATG transcription start site; the fragment was generated from the DNA template and cloned into a pGL4.10 vector with 5’ NheI and 3’HindIII restriction sequences. The deleted dual-luciferase plasmids pro-TIM3 A/B were purchased from GenePharma (China). The sgTOX and sgTOX2 plasmids were constructed by subcloning sgTOXRNA and sgTOX2RNA into the Crispr-Cas9 vector harboring a puromycin-resistance maker using the BsmBI site. shSirt1, shLCOR, and shhnRNPU plasmids were constructed using Sirt1-shRNA, LCOR-shRNA, and hnRNPU-shRNA cloned into pLKO.1 vector with 5’ AgeI and 3’ EcoRI endonuclease sequences. HEK293T cells were plated to 70-80% confluence in a 10 cm plate. Plasmid DNA (5 µg) was diluted in 500 µl OPTI-MEM, and then mixed with 20 µg of polyethyleneimine (PEI, Invitrogen, USA) for 15 min at room temperature. DNA and PEI solution was added dropwise to HEK293T cells for 24 h, and then replaced by the DMEM (Gibco) culture medium. Lentiviruses were generated by co-transfecting HEK293T cells with target plasmid. The packaging plasmids psPAX2 and pMD2.G were mixed at a 4:3:1 ratio with 64 µg of PEI diluted in 1000 µl OPTI-MEM, and then added dropwise to HEK293T cells for 8 h before replacing with fresh DMEM culture medium and culturing for 48 h. The lentivirus medium was collected and filtered through a 0.45-µm filter, and then centrifuged at 100,000 × *g* at 4 °C for 2 h using a high-speed refrigerated centrifuge. The pellet was re-suspended in 1 ml RPMI 1640 (Gibco) medium. Jurkat, MOLT3 and T cells were infected with targeting lentivirus plus 8 µg/ml polybrene (Sigma-Aldrich, USA). The plasmid vector, sgRNA, and shRNA sequences are provided in Supplementary Table 1.

### Antibody and reagent

Detailed information on the reagents and antibodies used in this study is provided in Supplementary Table 1.

### ChIP, quantitative real-time reverse transcription-PCR (qRT-PCR) analysis, luciferase reporter assays, and EMSAs

ChIP was performed using a ChIP Kit (Invitrogen, USA) according to the manufacturer’s instructions. Total RNA was extracted from cells using an RNA purification kit (EZBioscience, USA). cDNA was generated using a RevertAid First-Stand cDNA Synthesis Kit (Invitrogen, USA) and qPCR performed using the SYBR qPCR Master Mix (Tsingke Biotech, China) and CFX96 real-time PCR system (Bio-Rad, USA). Data were obtained as the relative abundance of mRNA normalized to the standard (i.e., GAPDH). ChIP-qPCR enrichment was calculated related to 1% input DNA. For luciferase reporter assays, HEK293T cells were seeded in 24-well plates for 24 h and co-transfected with TOX-TOX2, HAVCR2-promoter/HAVCR2-pro A/HAVCR2-pro B dual-luciferase plasmid (firefly luciferase, 500 ng) and pRL-TK (renilla luciferase, 50 ng) for 48 h. Luciferase activity was measured with the Dual-Luciferase Assay kit (Promega, USA) using the Microplate Luminometer (Promega Glomax96, USA) according to the manufacturer’s instructions. For EMSA, R1 (TOX motif1) and R9 (TOX motif9) probes (Supplementary Table 1) were labeled with biotin and EMSA performed using an EMSA kit (Beyotime, China) according to the manufacturer’s instructions.

### Immunofluorescence

Cells were plated in a confocal dish or on a slide treated with poly-D-lysine (Sigma- Aldrich, USA), and then fixed with 4% paraformaldehyde (Biosharp, China) for 15 min at room temperature. Cells were washed with PBS for 3 min three times and blocked using a sealed liquid (supplemented 0.3% Triton X-100) for 30 min with an Opal Polaris 7 Color manual IHC kit (Akoya Biosciences, USA) according to the manufacturer’s instructions. Coverslips were mounted onto slides with DAPI and the samples analyzed by high-resolution laser confocal microscopy (LSM880, ZEISS, Germany).

### Cell fractionation

Cytoplasmic and nuclear proteins were extracted using a cell fractionation kit (Cell Signaling Technology, USA) according to the manufacturer’s instructions. GAPDH or β-actin was used as an internal reference for cytoplasmic proteins, and histone H3 was used as an internal reference for nuclear proteins.

### Native-page

Nuclear and cytoplasmic protein samples were mixed with 0.5 µl of Native-page 5% G-250 Sample Additive (Thermo Scientific, USA) and subjected to electrophoresis on Tris-Gly gels (SDS free) at 4 °C. They were transferred electrophoretically to nitrocellulose membranes for immunoblotting.

### Co-immunoprecipitation, immunoprecipitation, chemical cross-linking, silver staining, and Western blotting

HEK293T cells (5 × 10^6^) were collected and rinsed three times with ice-cold PBS (Ph 7.4), and then resuspended in 200 µl PBS containing protease inhibitors. The cross-linking reagent EGS (Thermo Scientific, USA) was added to the cell suspension at a final concentration of 1 mM to incubate for 40 min at room temperature, and the reaction was stopped with Tris-HCl (1.5 M, Ph 8.8) for a final concentration of 20 mM. The quenched samples were mixed with 5X SDS loading buffer containing 50 mM DTT, incubated for 10 min at 70 °C, and then subjected to electrophoresis. For co-immunoprecipitation, cells were lysed with lysis buffer for 1 h on ice using a Co-IP kit (Thermo Scientific, USA), and lysates were incubated with primary antibodies for IP overnight at 4 °C. The target proteins were pulled down by protein A/G beads. For immunoprecipitation, T2, Jurkat, and HEK293T cells were fractionated, and the extracts were denatured at 100 °C for 10 min, immunoprecipitated using anti-TOX2 or anti-Flag, and immunoblotted using anti-phospho-ser. For silver staining, T cells were transfected with empty vector (EV) and TOX2-NLS-HA lentivirus and activated with OKT-3 for 3 days. Nuclear proteins were extracted from the EV and TOX2-NLS-HA group T cells and co-immunoprecipitation experiments performed using anti-TOX. The IP samples were subjected to electrophoresis and silver stained using a silver staining kit (Beyotime, China) according to the manufacturer’s instructions. For Western blotting, cell lysates or immunoprecipitates were separated by SDS–PAGE gels and then transferred to PVDF membrane (Millipore). Membranes were incubated with appropriate primary antibodies and horseradish peroxidase (HRP)-conjugated anti-rabbit/mouse secondary antibodies, and then visualized with the Bio-Rad system (Bio-Rad, Germany).

### Apoptosis assay and flow cytometry

pCDH/TIM3-OE/sgControl/sgTOX/sgTOX2/shNC/shLCOR-1 vector-transfected and DMSO/chidamide-treated Jurkat and MOLT3 cells were stained with PI and Annexin V (AF647) using an apoptosis assay kit (YiShan Biotech, China). Analysis was performed using a flow cytometer (CytoFLEX LX, Beckman, Germany).

### Bioinformatic analysis

The ChIP-seq data analysis was conducted using the bowtie2 and macs2 software packages. The data were sourced from the public database GSE93953, and HOMER was employed for the identification of transcription factor binding sites, motif prediction, and annotation of enriched regions. The heatmap package was used to visualize and cluster gene expression.

### Statistical analysis

The numerical data are presented as the mean ± SEM of three or more independent experiments and visualized using GraphPad Prism version 8.0. Unpaired two-tailed Student’s *t* test and one-way ANOVA were used for all statistical analyses. *P* < 0.05 between groups was considered significant.

## Supplementary information

Original Western Blot

Supplementary File1

SupplementaryFile 2

## Data Availability

Source data are provided in this paper. Information required for reanalyzing data from this paper is available from the corresponding author upon request. Source data are provided in this paper. The authenticity of this article has been validated by uploading the key raw data to the Research Data Deposit (RDD) public platform (www.researchdata .org.cn) under RDD approval number RDDB2024773292.
