# Supplementary material for: TOX2 nuclear-cytosol translocation is linked to leukemogenesis of acute T-cell leukemia by repressing TIM3 transcription
Source: Cell Death Differ. 2024 Jul 30;31(11):1506–18. doi: 10.1038/s41418-024-01352-z (PMC11519604; doi:10.1038/s41418-024-01352-z)
Supplement: Supplementary file 1 — Original Western Blot [file 41418_2024_1352_MOESM1_ESM.docx]

**Figure 1 D**


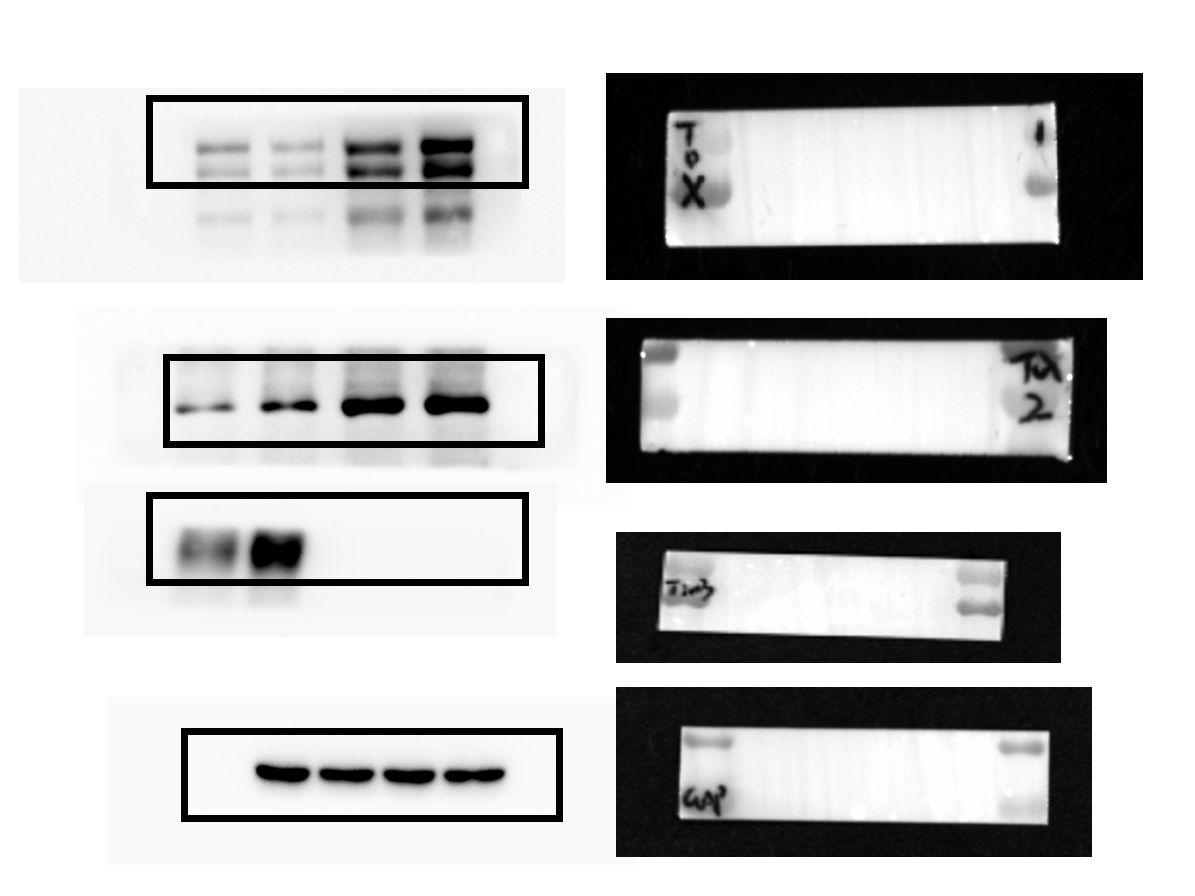


**Figure 2A**

**
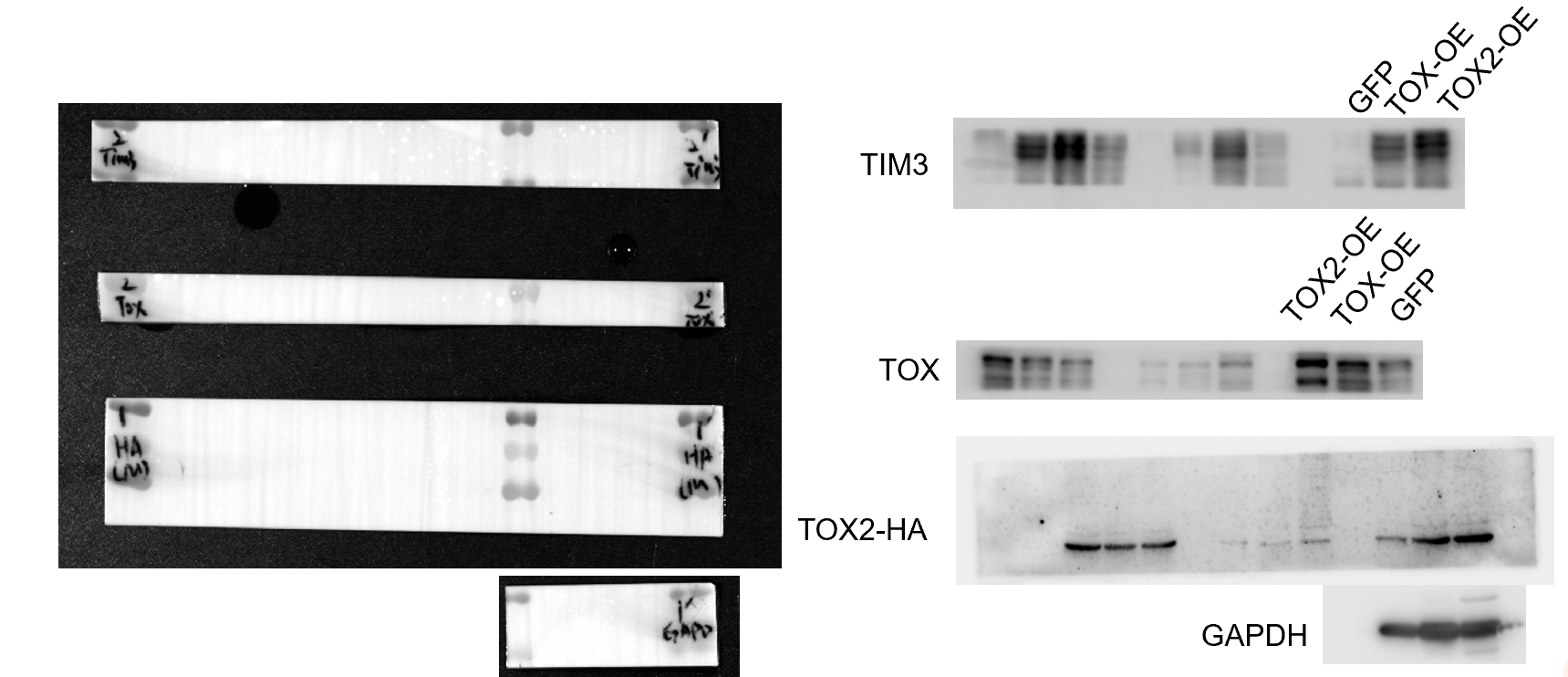
**

**Figure 2B**


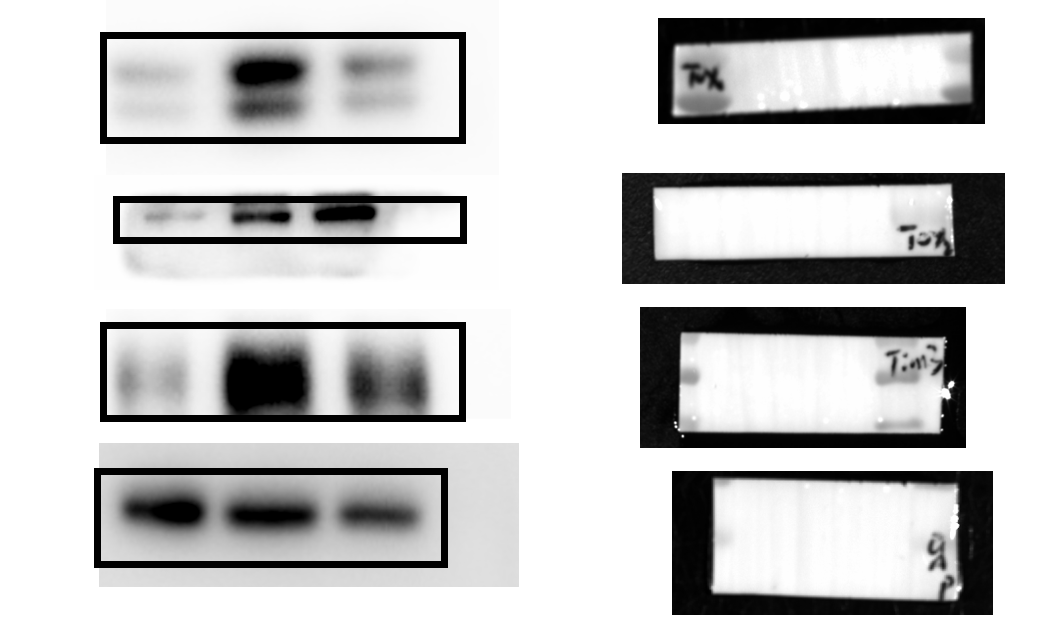


**Figure 2C**

**
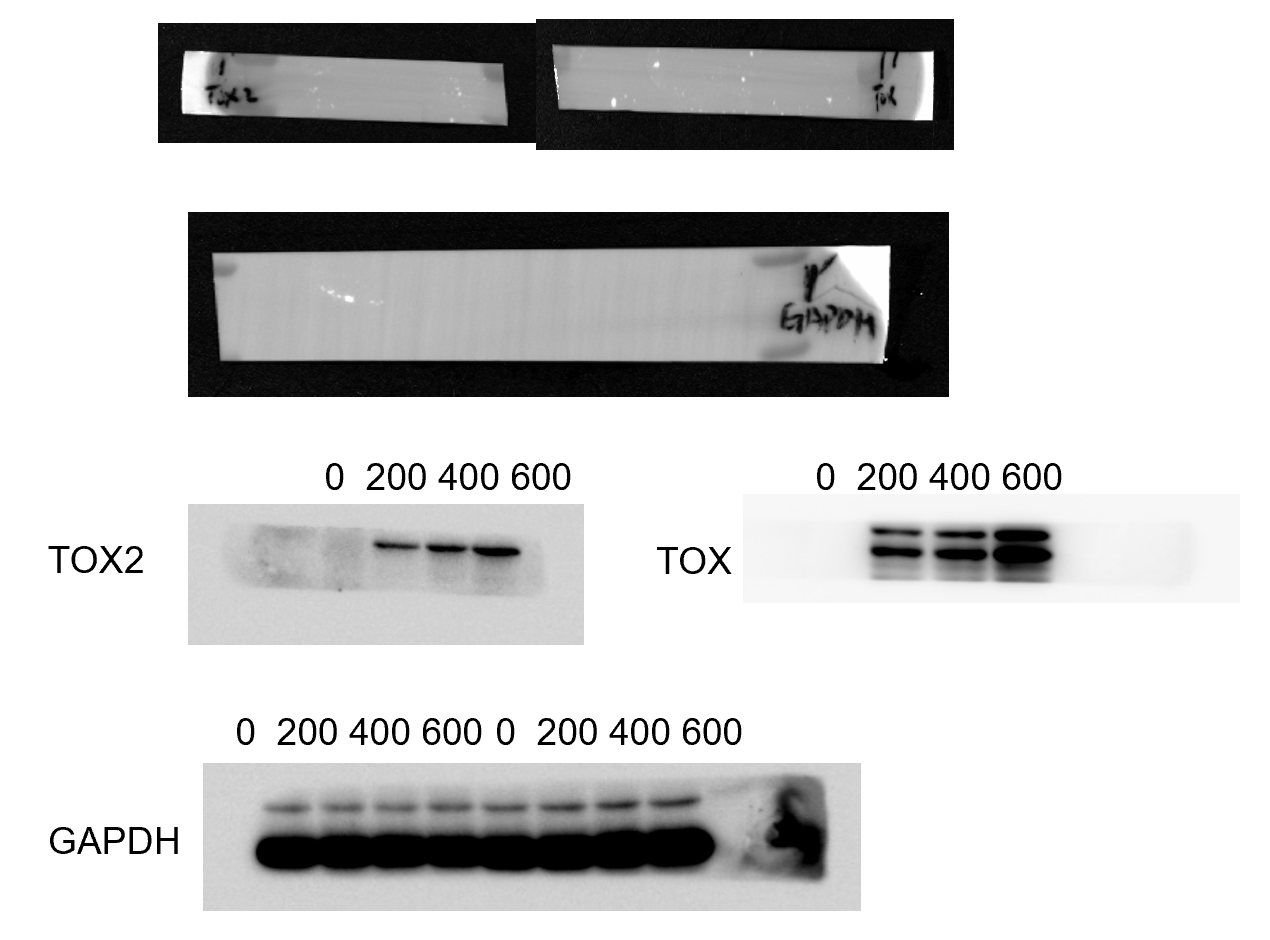
**

**Figure 3B**


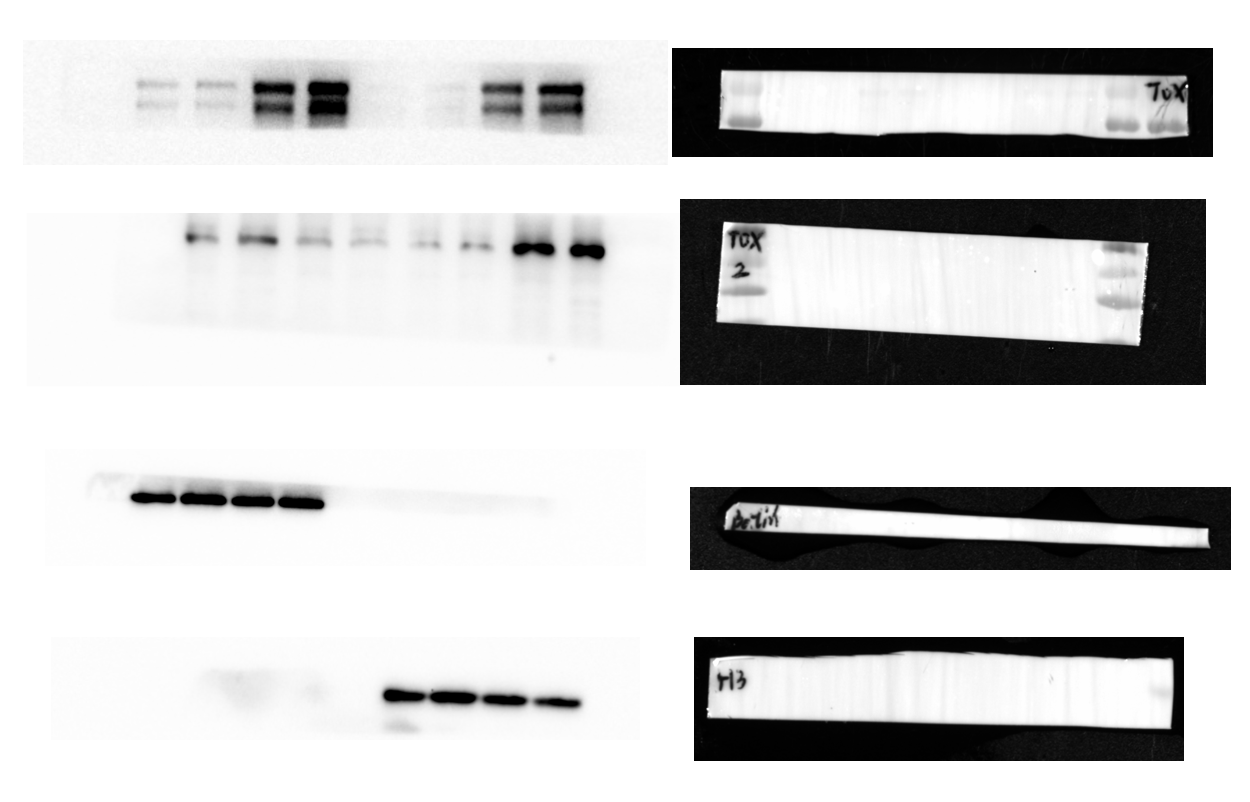


**Figure 3C**

**
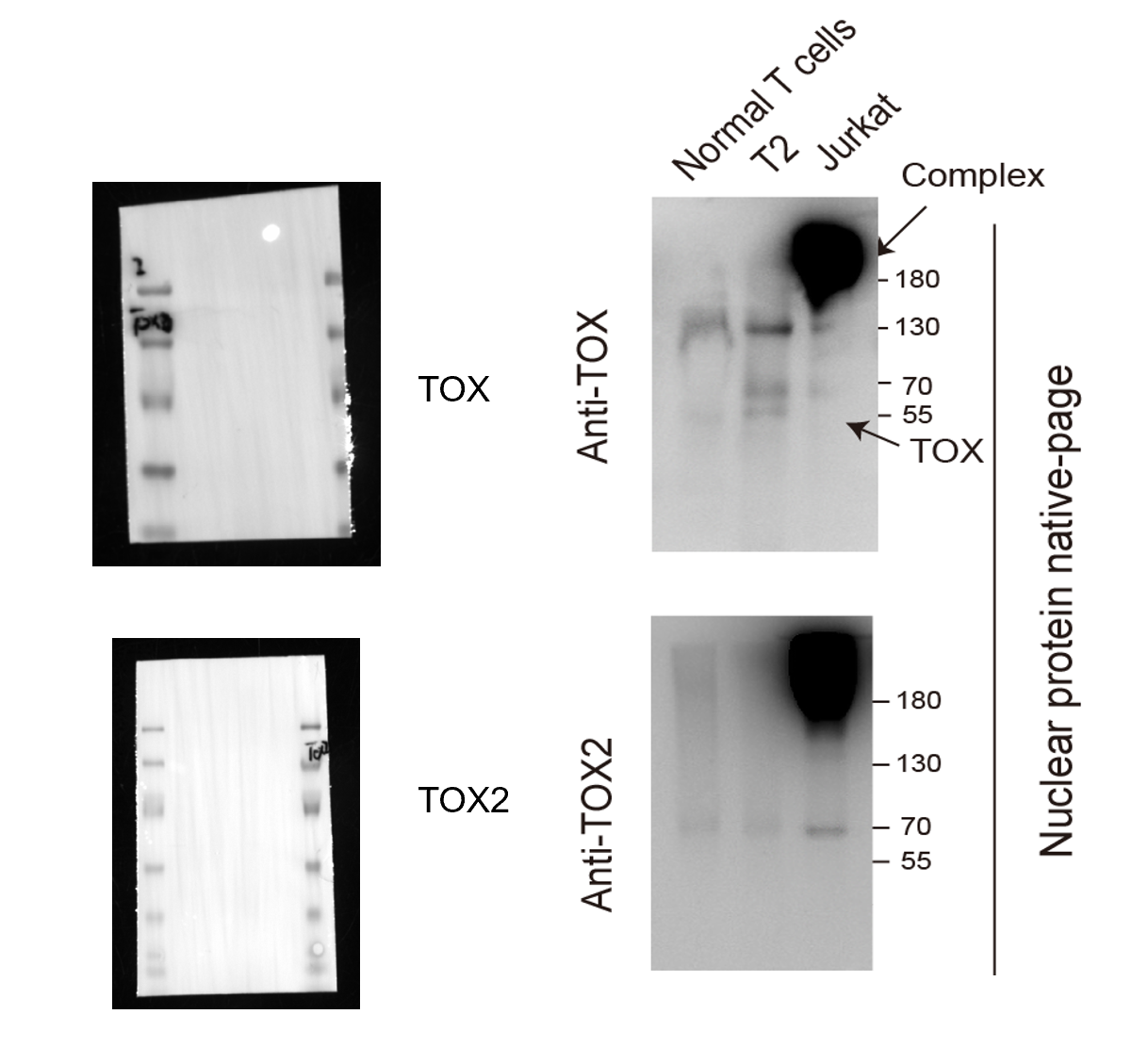
**

**Figure 3D**

**
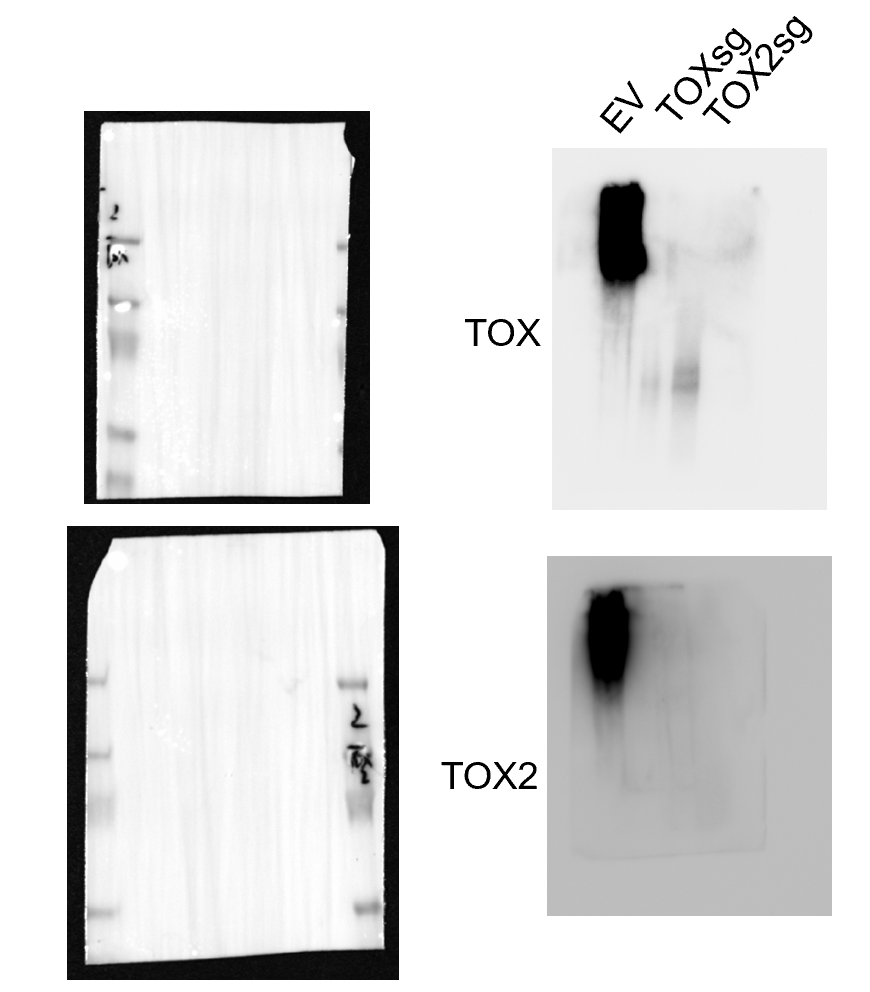
**

**Figure 3E**


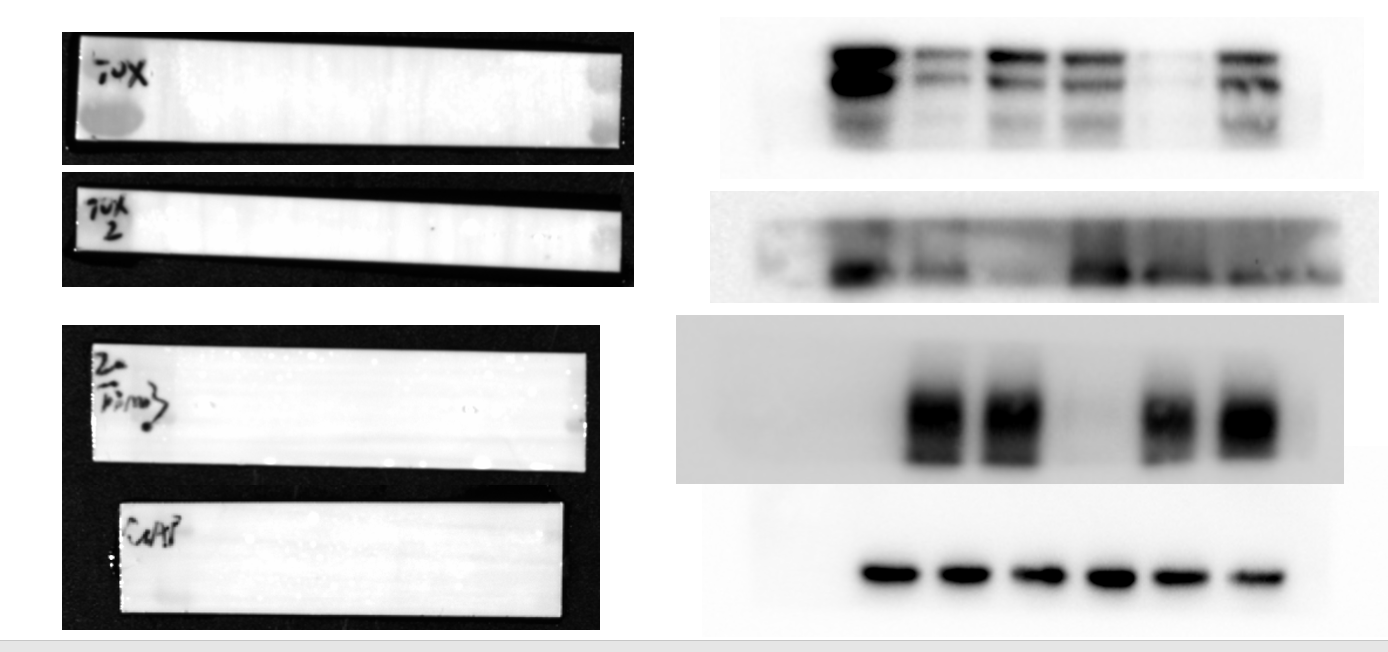


**Figure 3F**

**
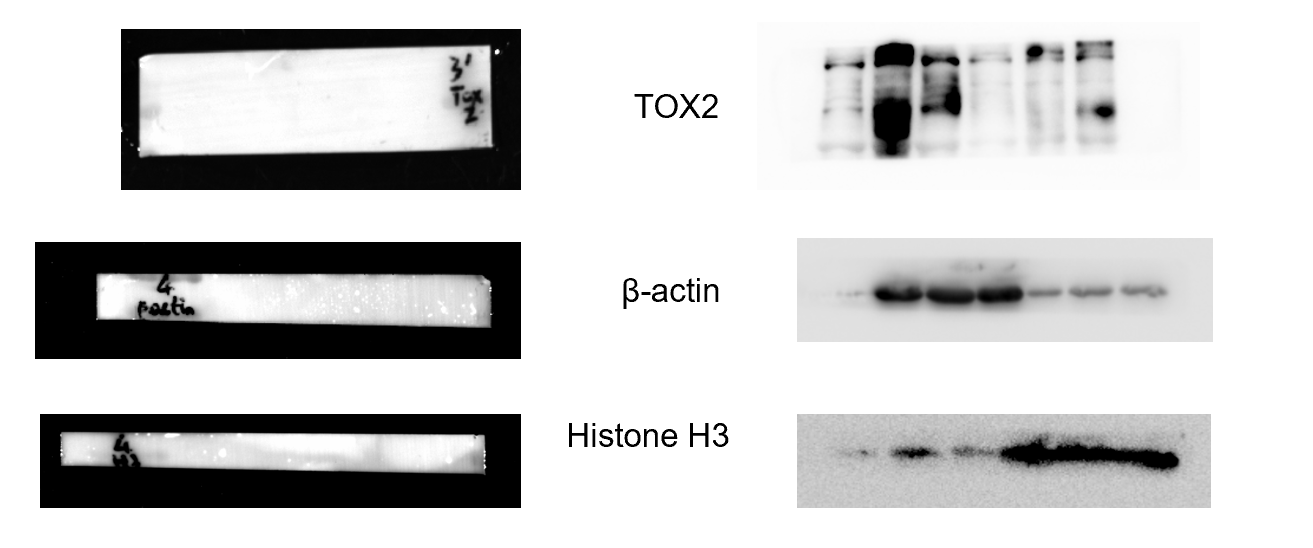
**

**Figure 3G**

**
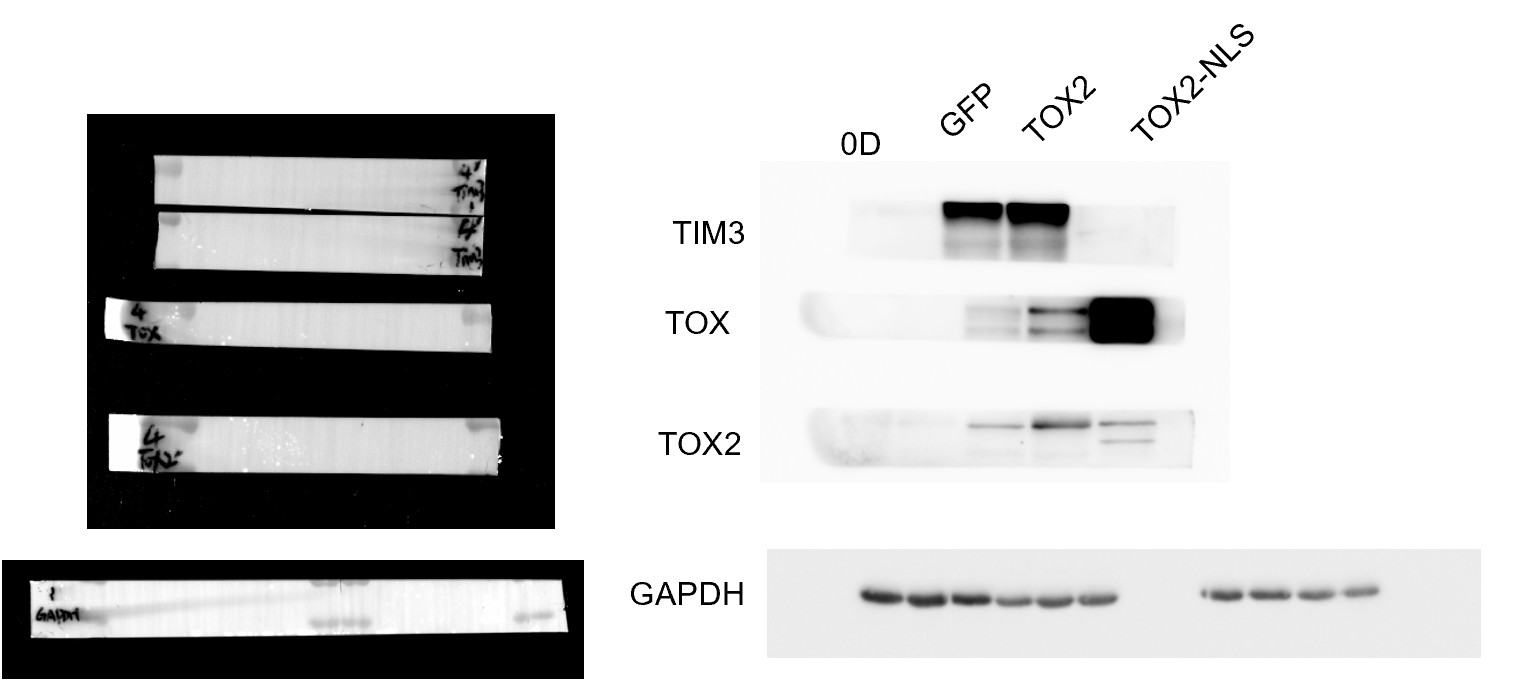
**

**Figure 3H**

**
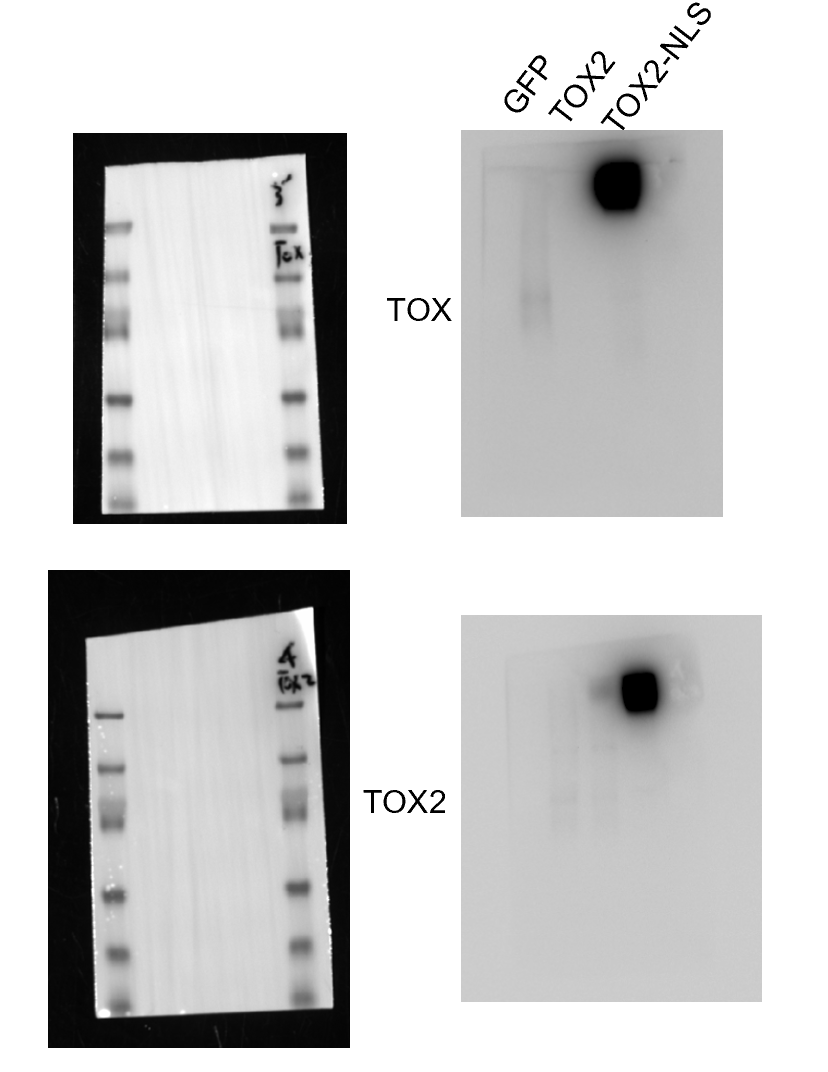
**

**Figure 4A(Left)**

**
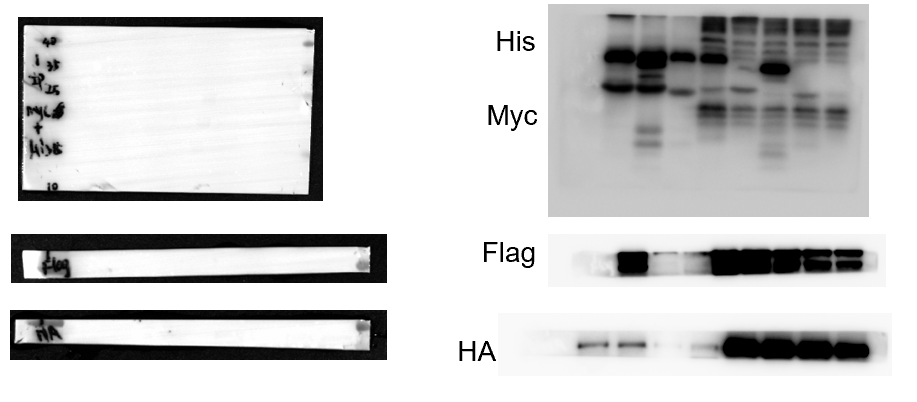
**

**Figure 4A（Right）**

**
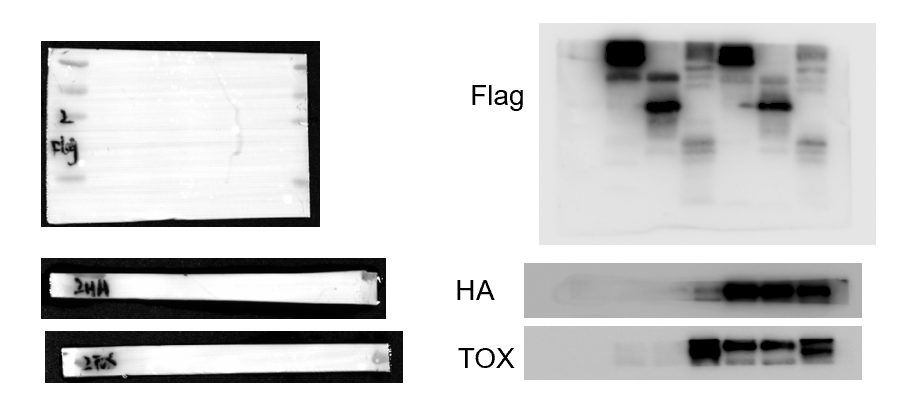
**

**Figure 4B**

**
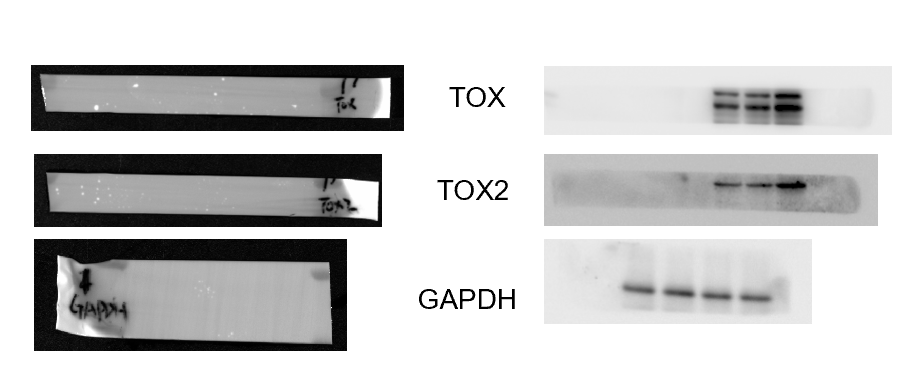
**

**Figure 4D**

**
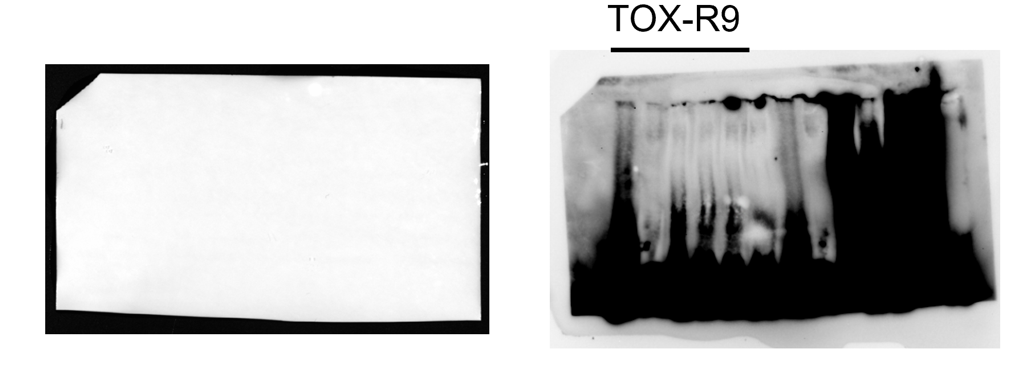
**

**
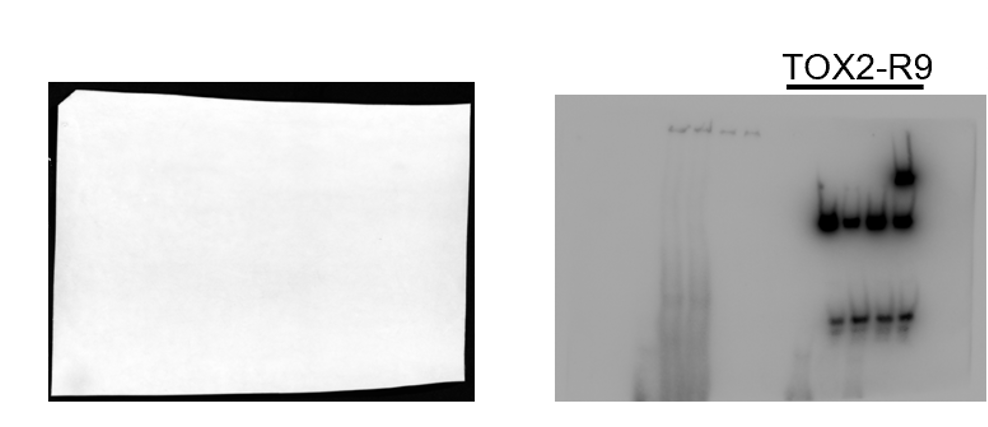
**

**
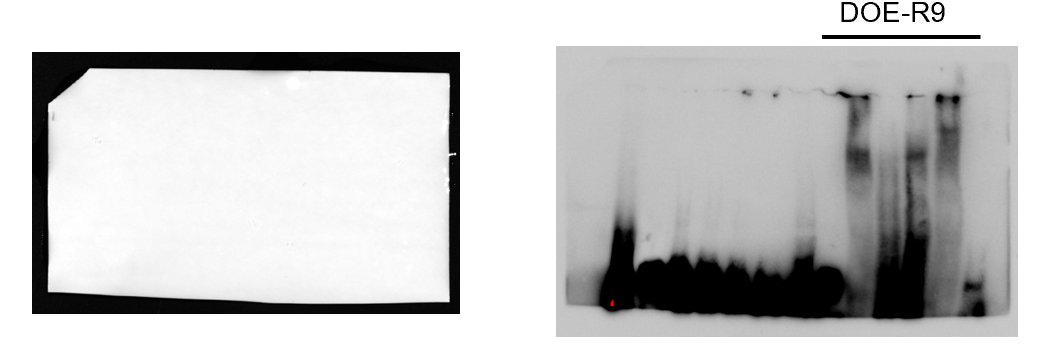
**

**
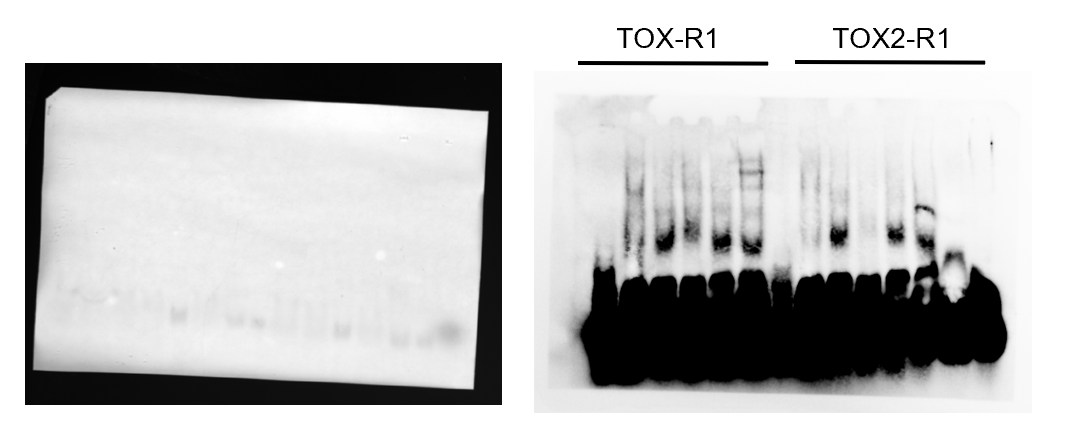
**

**
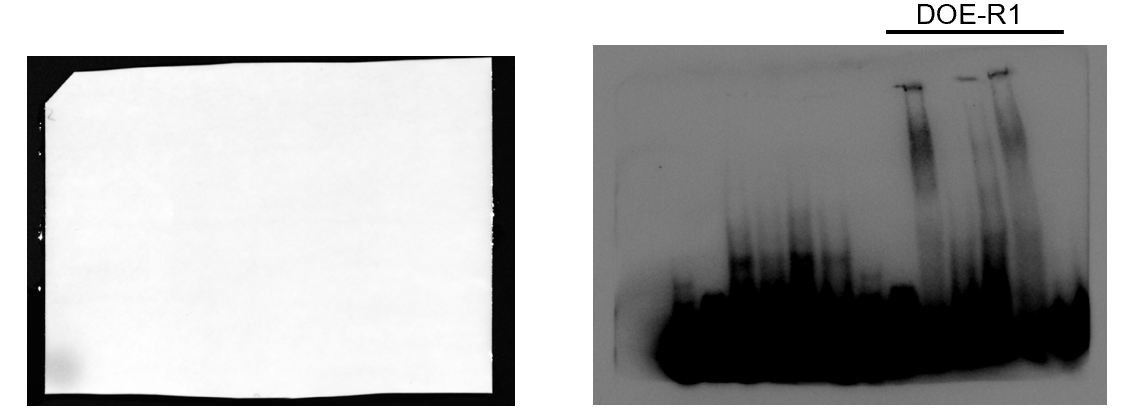
**

**Figure 5C**


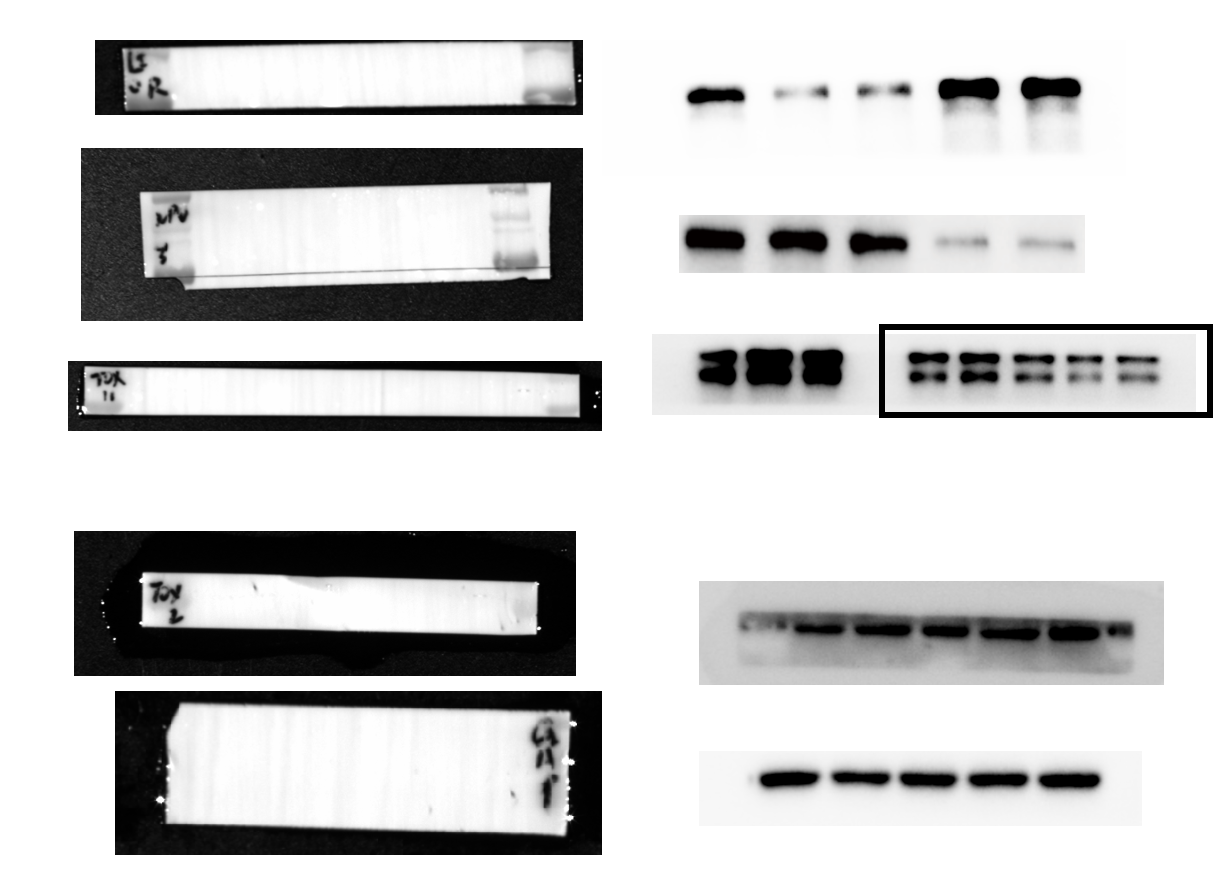


**Figure 5D**


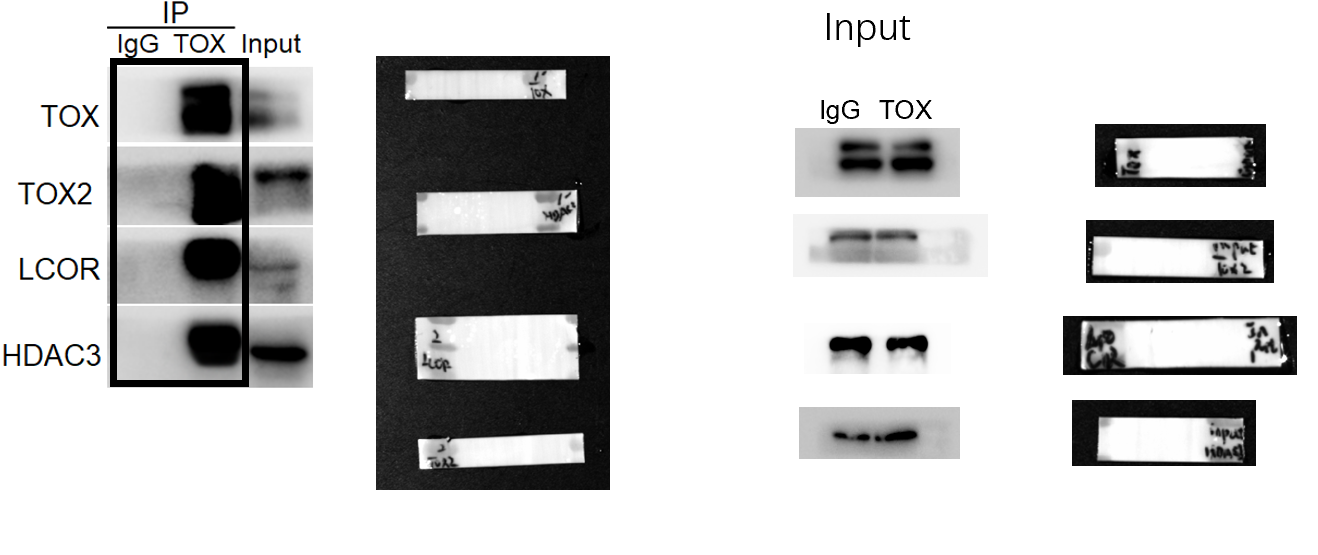


**Figure 5E**

**
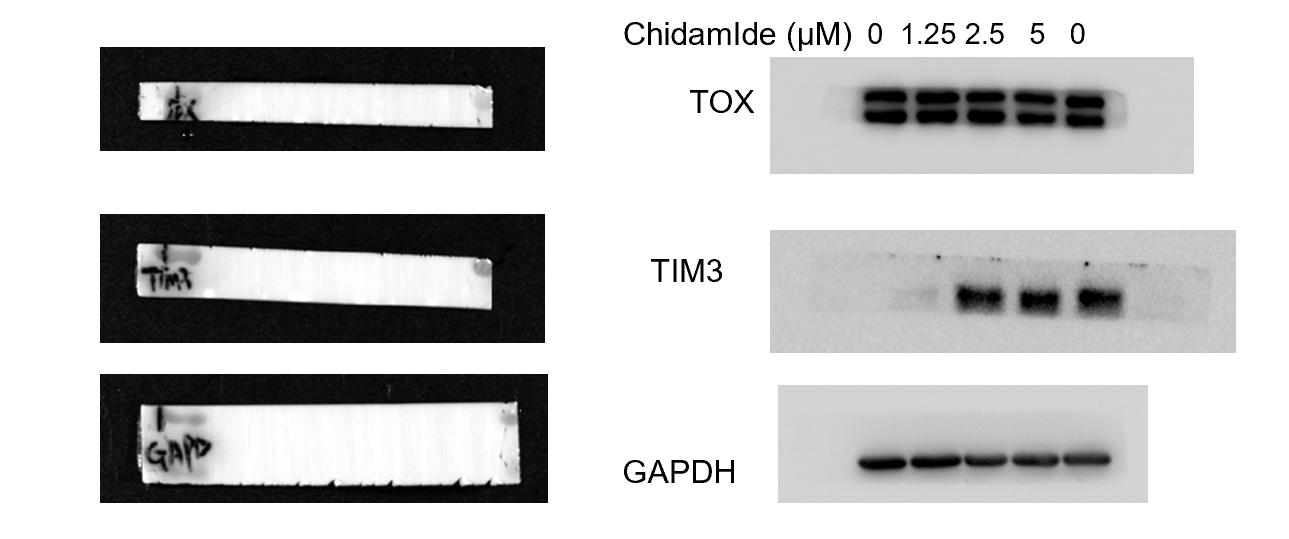
**

**Figure 6A**

**
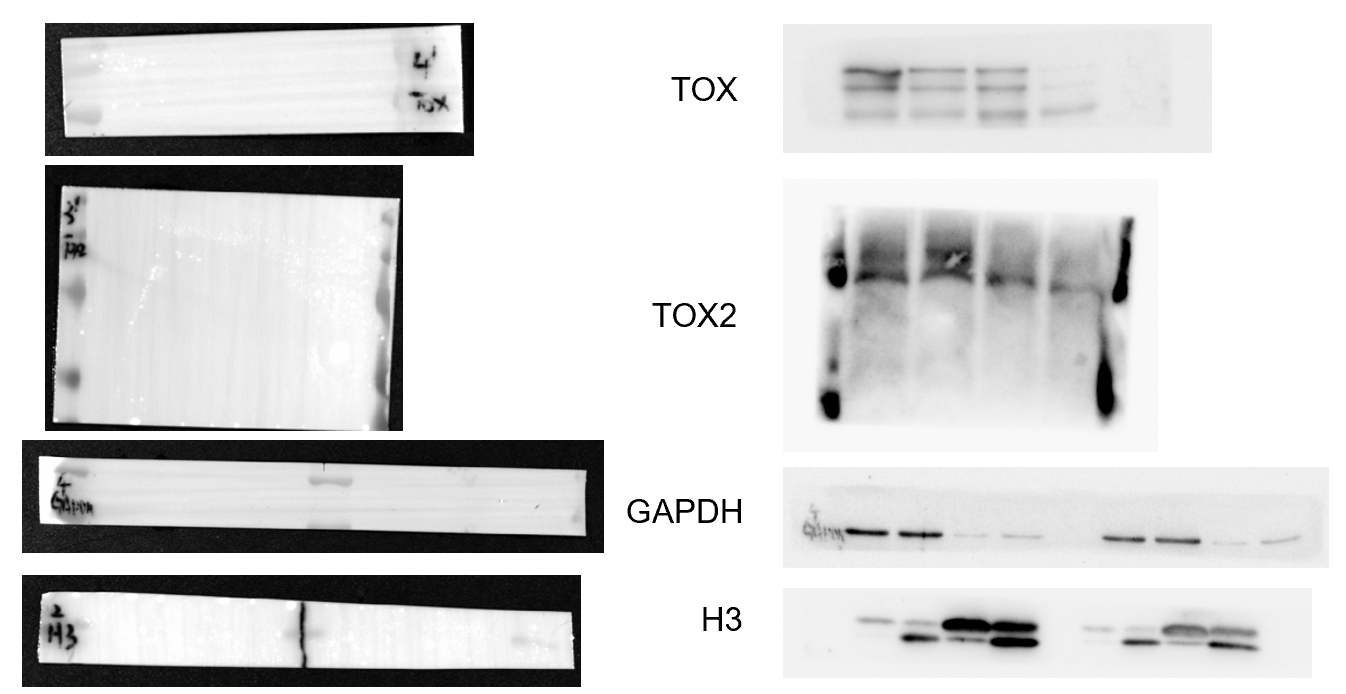
**

**Figure 6B**


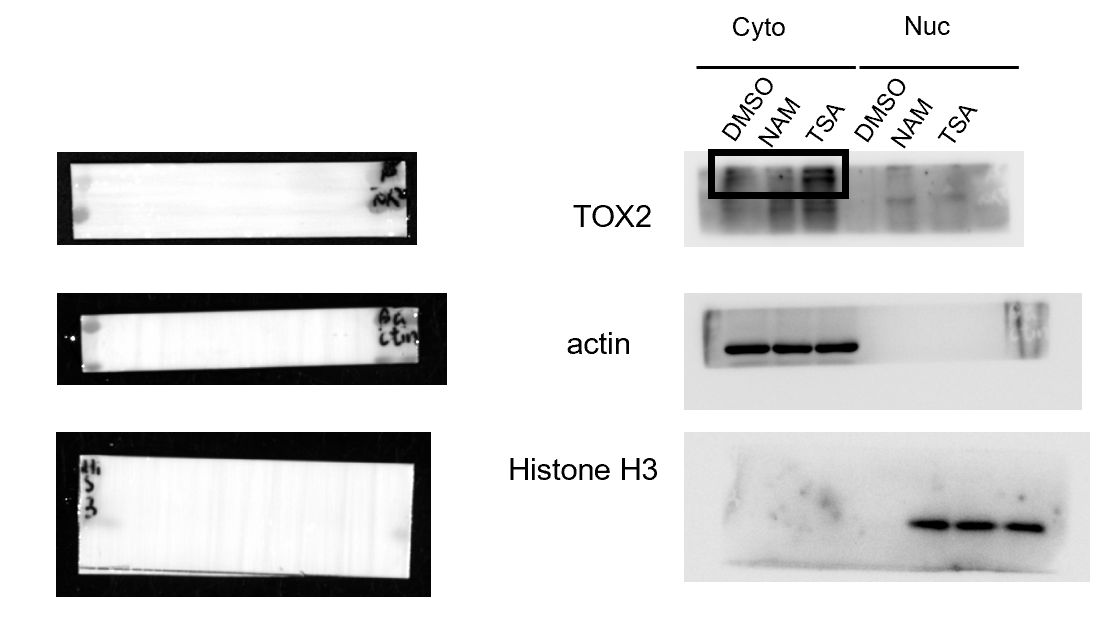


**Figure 6C**


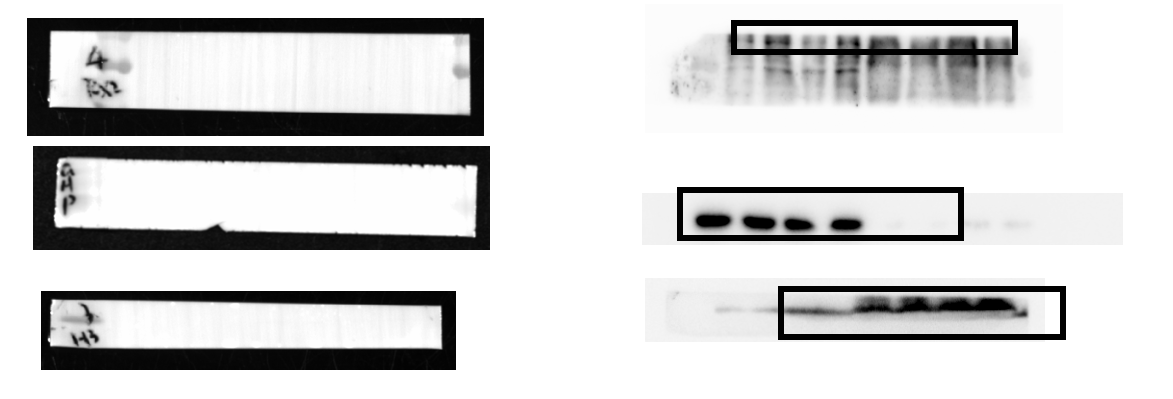


**Figure 6D**

**
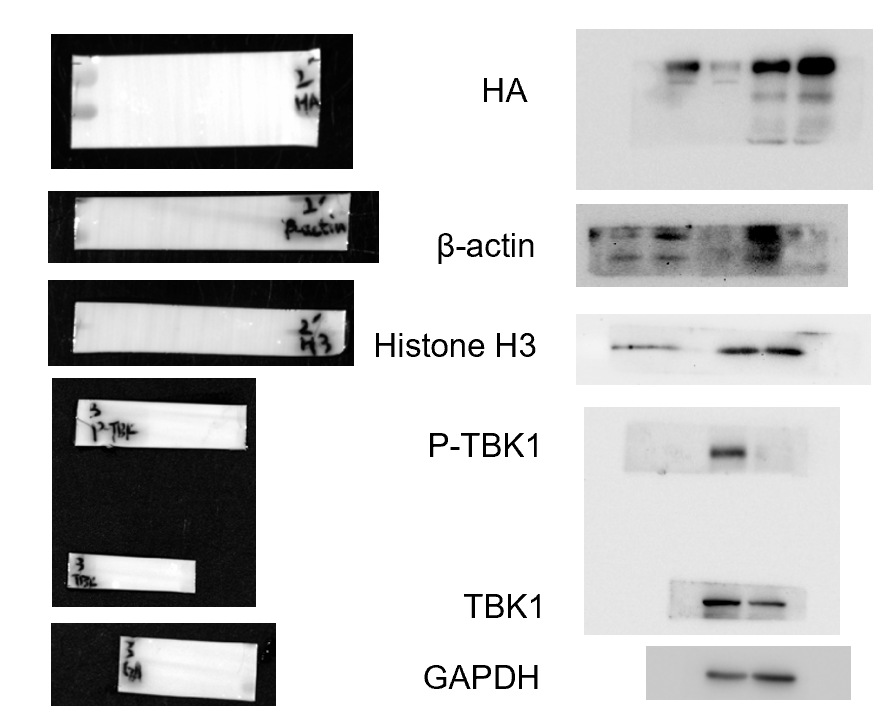
**

**Figure 6E**

**
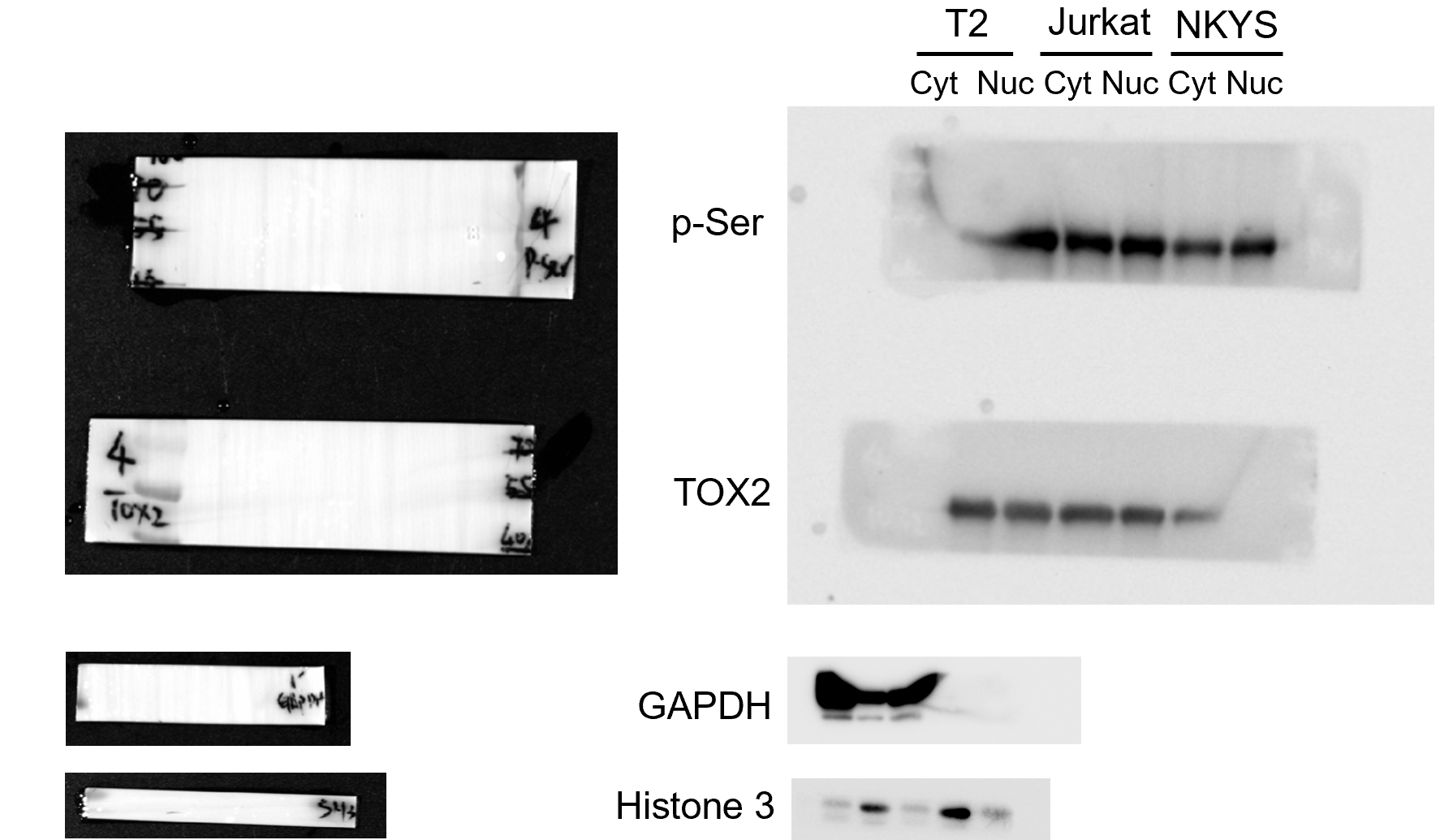
**

**Figure 6F**

**
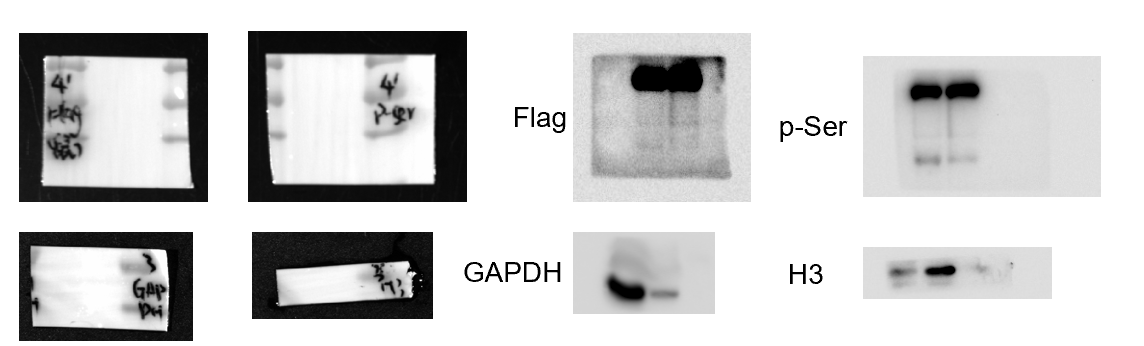
**

**Figure 6G**


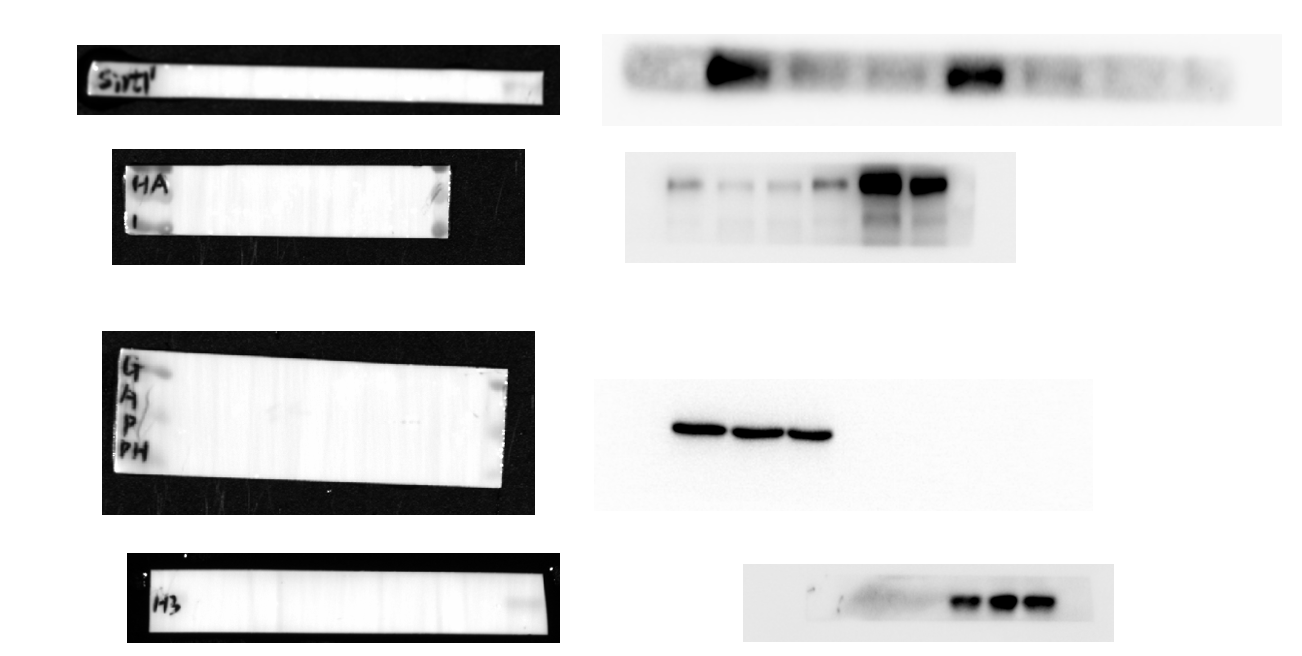


**Figure 6H**


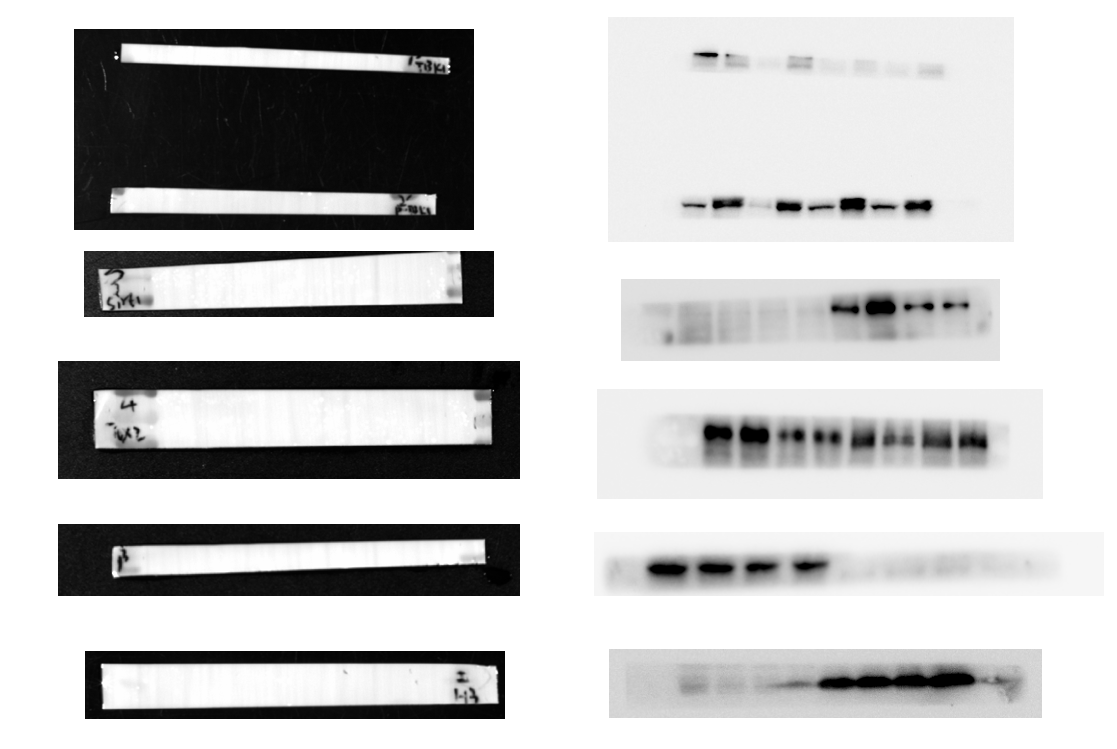


**Figure 6I**

**
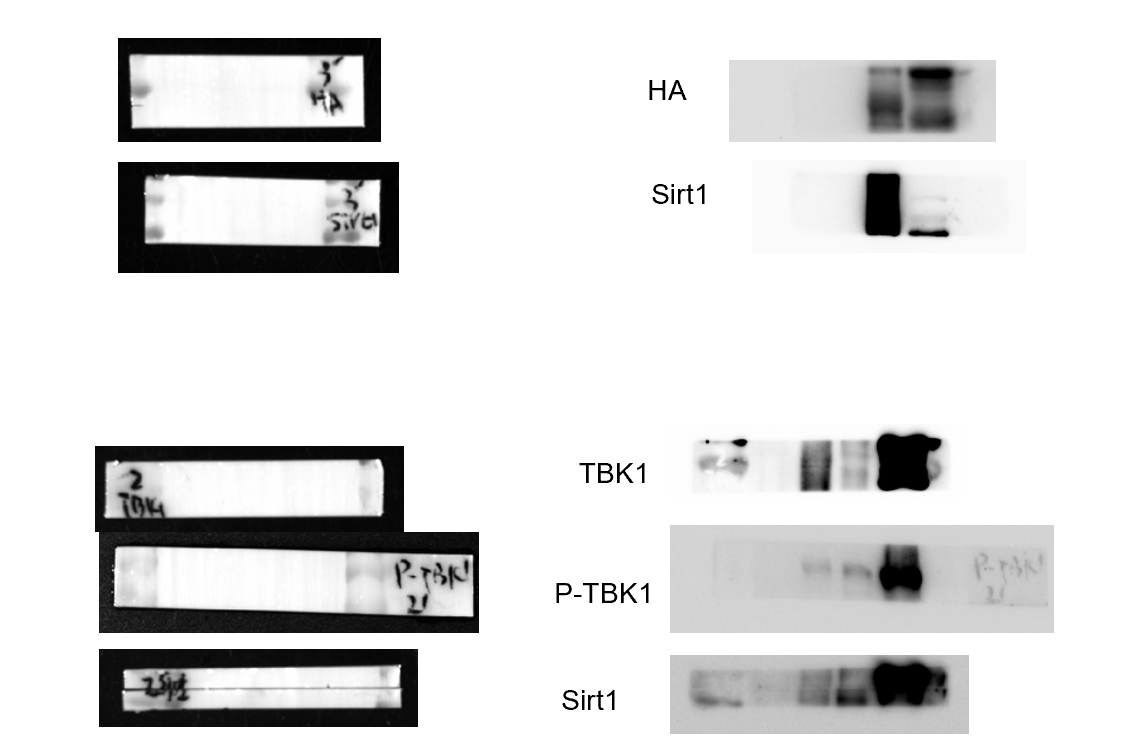
**

**Figure 6J**


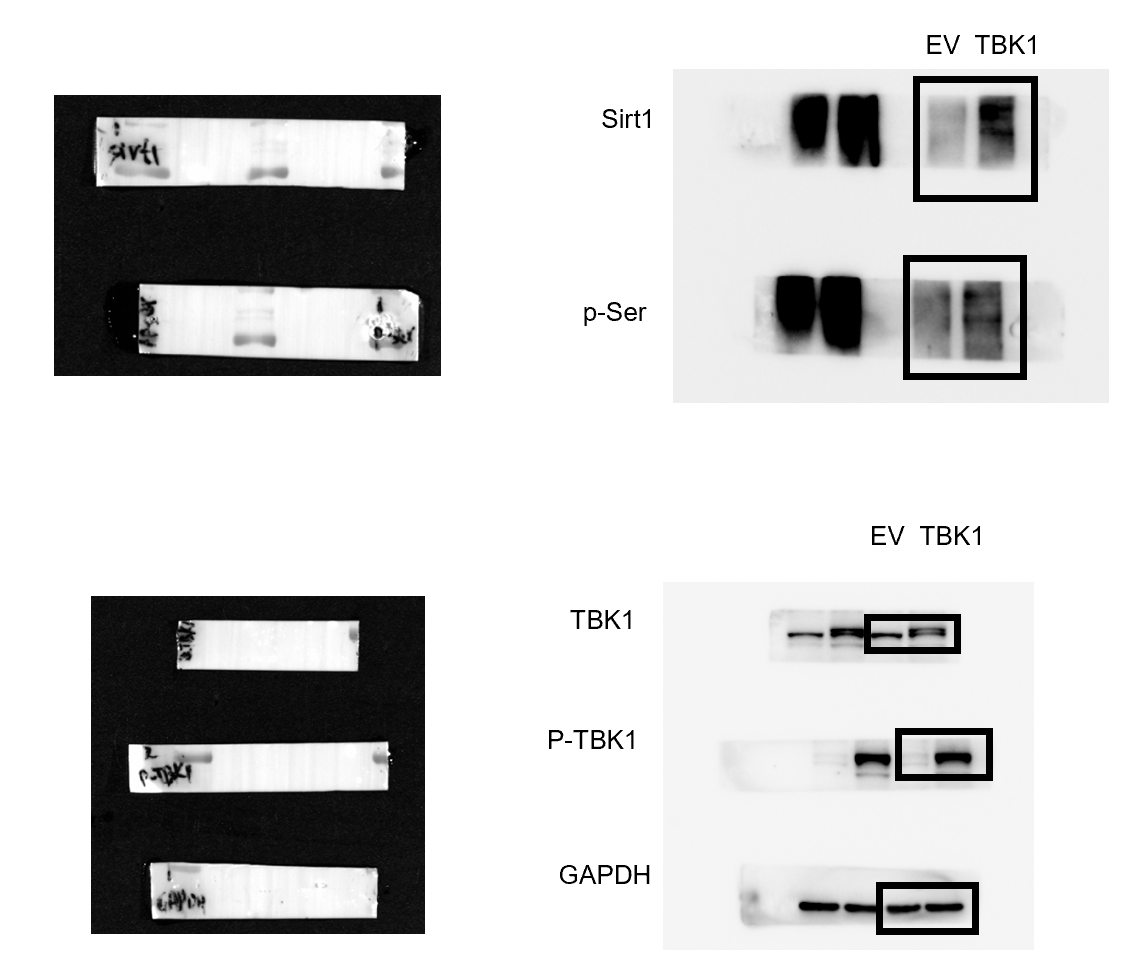


**Figure 6K**

**
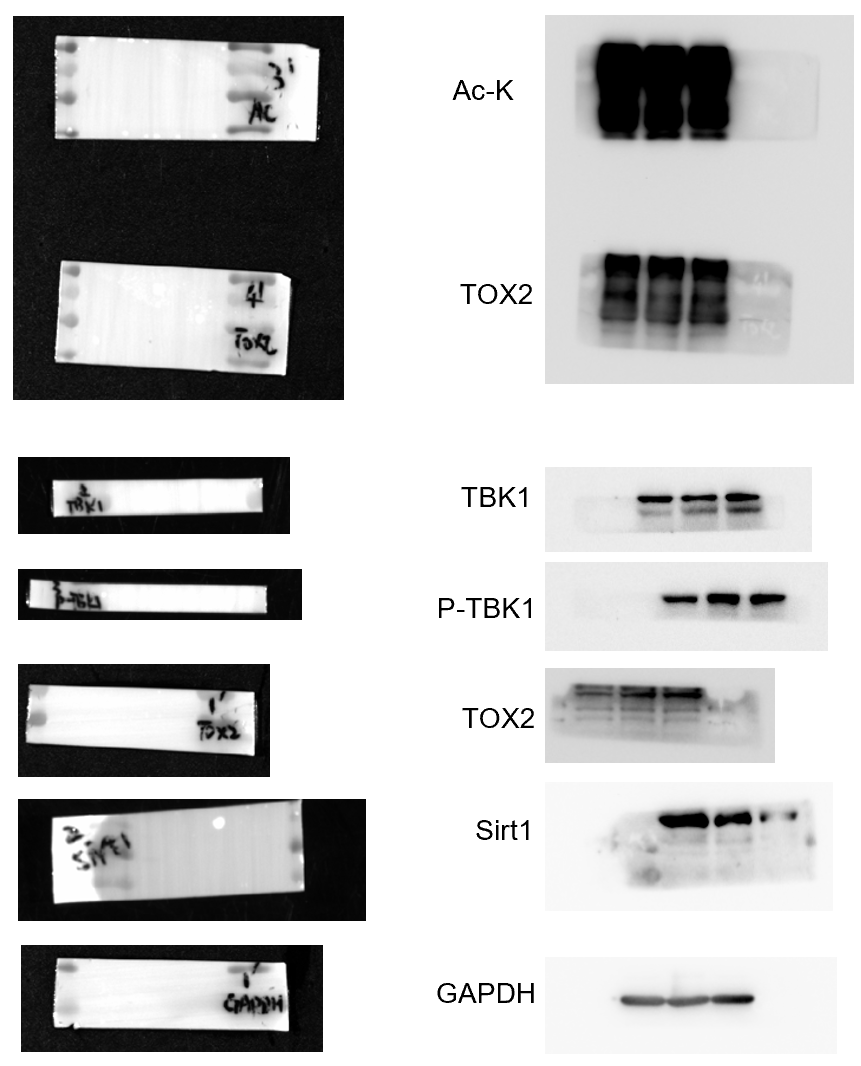
**

**Figure 7A**


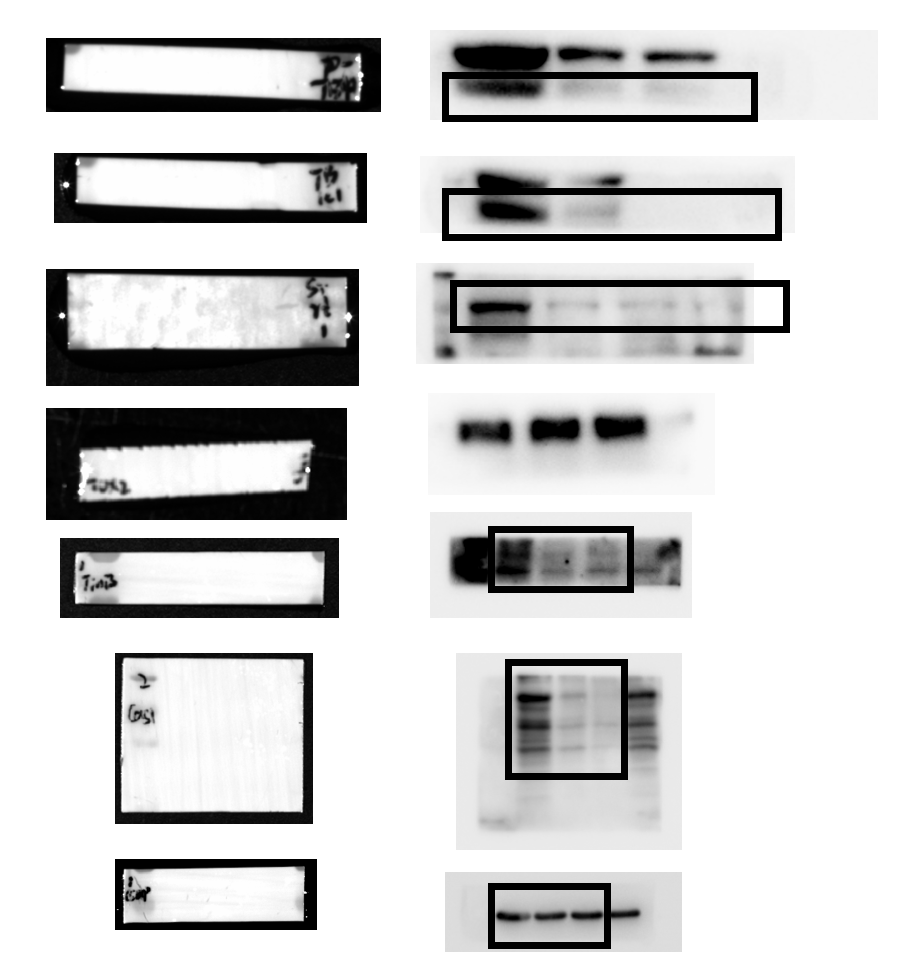


**Figure 7E**

**
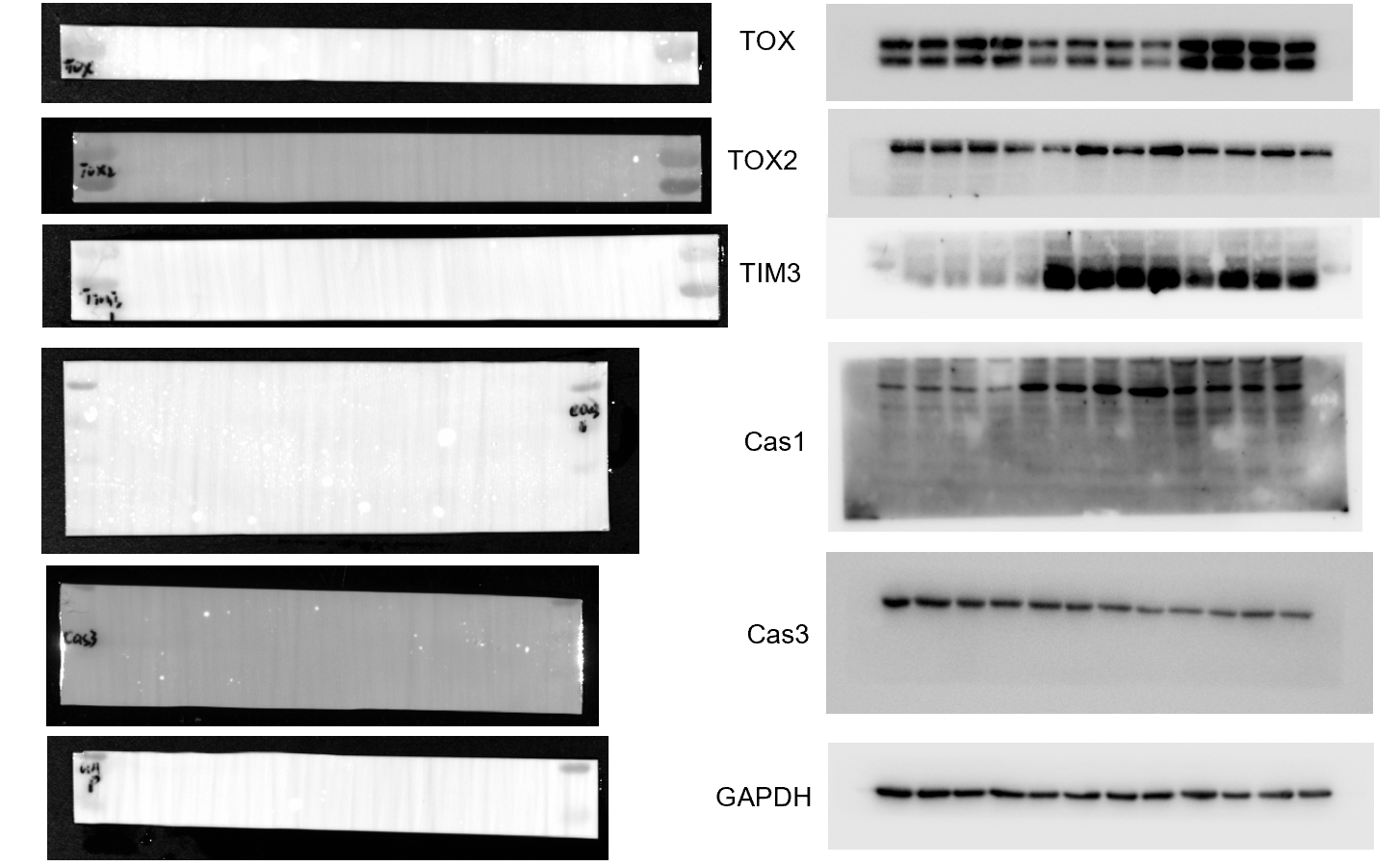
**
