# Supplementary material for: TOX2 nuclear-cytosol translocation is linked to leukemogenesis of acute T-cell leukemia by repressing TIM3 transcription
Source: Cell Death Differ. 2024 Jul 30;31(11):1506–18. doi: 10.1038/s41418-024-01352-z (PMC11519604; doi:10.1038/s41418-024-01352-z)
Supplement: Supplementary file 2 — Supplementary File1 [file 41418_2024_1352_MOESM2_ESM.docx]

**Supplementary table 1. Reagents, primers, and probes**

| **Reagent** | **Source and identifier** |
| --- | --- |
| Anti-TOX | CST (Cat# 36778S) |
| Anti-TOX2 | Proteintech (Cat# 21162-1-AP) |
| Anti-TIM3 | Abcam (Cat# ab241332) |
| Anti-normal rabbit IgG | CST (Cat# 2729S) |
| Anti-Flag | CST (Cat# 8146S) |
| Anti-HA | CST (Cat# 3724S) |
| Anti-His | CST (Cat# 2366S) |
| Anti-TBK1 | CST (Cat# 3504S) |
| Anti-p-TBK1 | CST (Cat# 5483S) |
| Anti-Sirt1 | Abclonal (Cat#A19667) |
| Anti-LCOR | SantaCruz (Cat# sc-377019) |
| Anti-HDAC3 | SantaCruz (Cat# sc-376957) |
| Anti-phosphoserine | Merck Millipore (Cat# AB1603) |
| Anti-GAPDH | Proteintech (Cat# 60004-1-Ig) |
| Anti-beta (β)-actin | Proteintech (Cat# 20536-1-AP) |
| Anti-histone H3 | CST (Cat# 5192S) |
| Anti-HA | CST (Cat# 2367S) |
| Anti-Myc | CST (Cat# 2276S) |
| Anti-acetylated-lysine | CST (Cat# 9814S) |
| Anti-caspase 1 | Proteintech (Cat# 22915-1-AP) |
| Anti-caspase 3 | Proteintech (Cat# 19677-1-AP) |
| Anti-human TIM3 Brilliant Violet510 | Biolegend (Cat# 345030) |
| RevertAid First-Stand cDNA Synthesis Kit | ThermoFisher (Cat# K1622) |
| RNA purification Kit | EZBioscience (Cat# B0004D) |
| ChIP Kit | Invitrogen (Cat# 26157) |
| SYBR qPCR Master Mix | Tsingke (Cat# TSE501) |
| PEI | Polysciences (Cat# 23966) |
| DMEM | Gibco (Cat# C11995500BT) |
| RPMI 1640 | Gibco (Cat# C11875500BT) |
| Polybrene | Sigma-Aldrich (Cat# TR-1003-G) |
| Opal Polaris 7 Color manual IHC Kit | Akoya (Cat# NEL861001KT) |
| Cell fractionation kit | CST (Cat# 9038S) |
| Nativepage 5% G-250 Sample Additive | Invitrogen (Cat# BN2004) |
| EGS | ThermoFisher (Cat# 21565) |
| EMSA kit | Beyotime (Cat# GS009) |
| Poly-D-lysine | Sigma-Aldrich (Cat# P1149) |
| Silver staining kit | Beyotime (Cat# P0017S) |
| Chidamide | MCE (Cat# HY-109015) |
| Apoptosis assay kit | Elabscience (Cat# E-CK-A213) |
| Matrix gel | Beyotime (Cat# C0383) |
| **qPCR primer** | **Primer sequence** |
| TOX-F | GTGATGCCAGATATACGAAACCC |
| TOX-R | AGCTGTGACTGGTTAATGGTAGT |
| TOX2-F | AGTCGGAAGTGCATTTCAAGAT |
| TOX2-R | GGCCTGAGTGTCTCTGAAGA |
| TIM3-F | GGAATACAGAGCGGAGGTCG |
| TIM3-R | CACCACGTTGCCACATTCAA |
| GAPDH-F | CGAGATCCCTCCAAAATCAAGTGGGG |
| GAPDH-R | ACACGTTGGCAGTGGGGACAC |
| Sirt1-F | TGGGTACCGAGATAACCTTCTGT |
| Sirt1-R | GTTCGAGGATCTGTGCCAATCATA |
| LCOR-F | CCAATTGTCCACAGCTGCCA |
| LCOR-R | TGCTGAGTGCACCATTTCCC |
| hnRNPU-F | GGTGGAATGCCCAACAGAGG |
| hnRNPU-R | ACCCCAGAATTGACCCTGCT |
| **RNA interfering primer** | **Primer sequence** |
| TOXsgRNA-F | GCTGATCAATTCGAAGCCGT |
| TOXsgRNA-R | ACGGCTTCGAATTGATCAGC |
| TOX2sgRNA-F | GACTTGCTCCCCGGCGGTGAT |
| TOX2sgRNA-R | ATCACCGCCGGGGAGCAAGTC |
| TBK1sgRNA-F | GCATAAGCTTCCTTCGTCCAG |
| TBK1sgRNA-R  shTOX-1  shTOX-2  shTOX2-1  shTOX2-2  shLCOR-1  shLCOR-2 | CTGGACGAAGGAAGCTTATGC  CCCTGAAATCACAGTCTCCAA  CGACTATCAGACTATTATCAA  AGCGAGAACAACGAAGACTAT  CAAATCGCTCTACCTCACCTA  CCAGATGTTTCTGTAAAGATT  CCAGCCCAATAGCACAAAGAA |
| shSirt1-1 | CAGGTCAAGGGATGGTATTTA |
| shSirt1-2 | CATGAAGTGCCTCAGATATTA |
| shhnRNPU-1 | CAGTGCTTCTTCCCTTACAAT |
| shhnRNPU-2 | GCAACTGTGAGACTGAAGATT |
| **Primer on TIM3** | **Primer sequence promoter for ChIP qPCR** |
| TIM3-pro-R1-F | TGGTGGTGTGCGCTCTTAGTC |
| TIM3-pro-R1-R | GCTGGAGTGCAGTGGCGC |
| TIM3-pro-R2-F | ATCTGTCACTTAGGGGGCTTATG |
| TIM3-pro-R2-R | GCCCGGCCAATTTCTCAT |
| TIM3-pro-R3-F | CAAATTTGGTATTTATACCCAATGC |
| TIM3-pro-R3-R | CAGTGGCCTTCCTCTATGTCTTG |
| TIM3-pro-R4-F | TCAGCCTGTGAGCTCACTTCC |
| TIM3-pro-R4-R | CCACACTCCCATAACTGAGGTAAC |
| TIM3-pro-R5-F | GCCAAAGCCTCTTGGGGTAG |
| TIM3-pro-R5-R | TTGGCAACAGTTCCTTTCCTCTAC |
| TIM3-pro-R6-F | GGAACTGTTGCCAAAAGGAACA |
| TIM3-pro-R6-R | CTTGCTGAGTCTCAGTTTCCTCAT |
| TIM3-pro-R7-F | GCTGGGAGTTGCTATGGTCTG |
| TIM3-pro-R7-R | TTCATCAGGCCTGTGACCAA |
| TIM3-pro-R8-F | GCTAGTCAGATGGACTCGGACA |
| TIM3-pro-R8-R | CAGTCATGGCACATAAAGAGAACC |
| TIM3-pro-R9-F | TGTTGATATCACAGGACAGACATCA |
| TIM3-pro-R9-R | CCAGGTCTACAGTCACATTAAAGGA |
| TIM3-pro-R10-F | AAGAATCACTGGCAATCAGACAC |
| TIM3-pro-R10-R | GTCTACTCCACAATCACATGAGCA |
| **Probe** | **Probe sequence** |
| Probe-R1 | CACAGGAGGCGGAGTTTGCAGTGAGCC  GAGATCGCGCCACTGCACTCCAGCCTG  GGCGAC |
| Probe-R9 | TTACAGGATGTGTGTAGTGTGGCATGAC  AGAGAACTTTGGTTTCCTTTAATGTGAC  TGTA |
| Mutant probe R1 | CACAAGCCGAGATCGCGCCACTGCACTC  CAGCCTGGGCGAC |
| Mutant probe R9 | TTACAGGGCATGACAGAGAACTTTCCTTT  AATGTGACTGTA |
| **Nucleoplasmin** | **Sequence** |
| NLS (Nuclear localization signal) | AAGCGTCCTGCTGCTACTAAGAAAGCT  GGTCAAGCTAAGAAAAAGAAA |

**Supplementary table 2.** **Abbreviations**

| **Abbreviation** | | | **Full term** | |
| --- | --- | --- | --- | --- |
| TOX | Thymocyte selection-associated high mobility group box | | |  |
| TOX2 | | TOX high mobility group box family member 2 | | |
| TIM3 | | T-cell immunoglobulin mucin family member 3 | | |
| GFP | | Green fluorescent protein | | |
| NLS | | Nuclear localization signal | | |
| Sirt1 | | Sirtuin 1 | | |
| Co-IP | | Co-immunoprecipitation | | |
| ChIP | | Chromatin immunoprecipitation | | |
| qPCR | | Quantitative real-time polymerase chain reaction | | |
| DAPI | | 4', 6-diamidino-2phenylindole | | |
| EMSA | | Electrophoretic mobility shift assay | | |
| LCOR | | Ligand-dependent corepressor | | |
| HDAC3 | | Histone deacetylase 3 | | |
| TBK1 | | TANK-binding kinase 1 | | |
| p-TBK1 | | Phosphorylated TBK1 | | |
| hnRNPU | | Heterogeneous nuclear ribonucleoprotein U | | |
| NAM | | Nicotinamide | | |
| TSA | | Trichostatin A | | |
| Ac-K | | Acetylated lysine | | |
| EV | | Empty vector | | |

**Supplementary Figure legend**


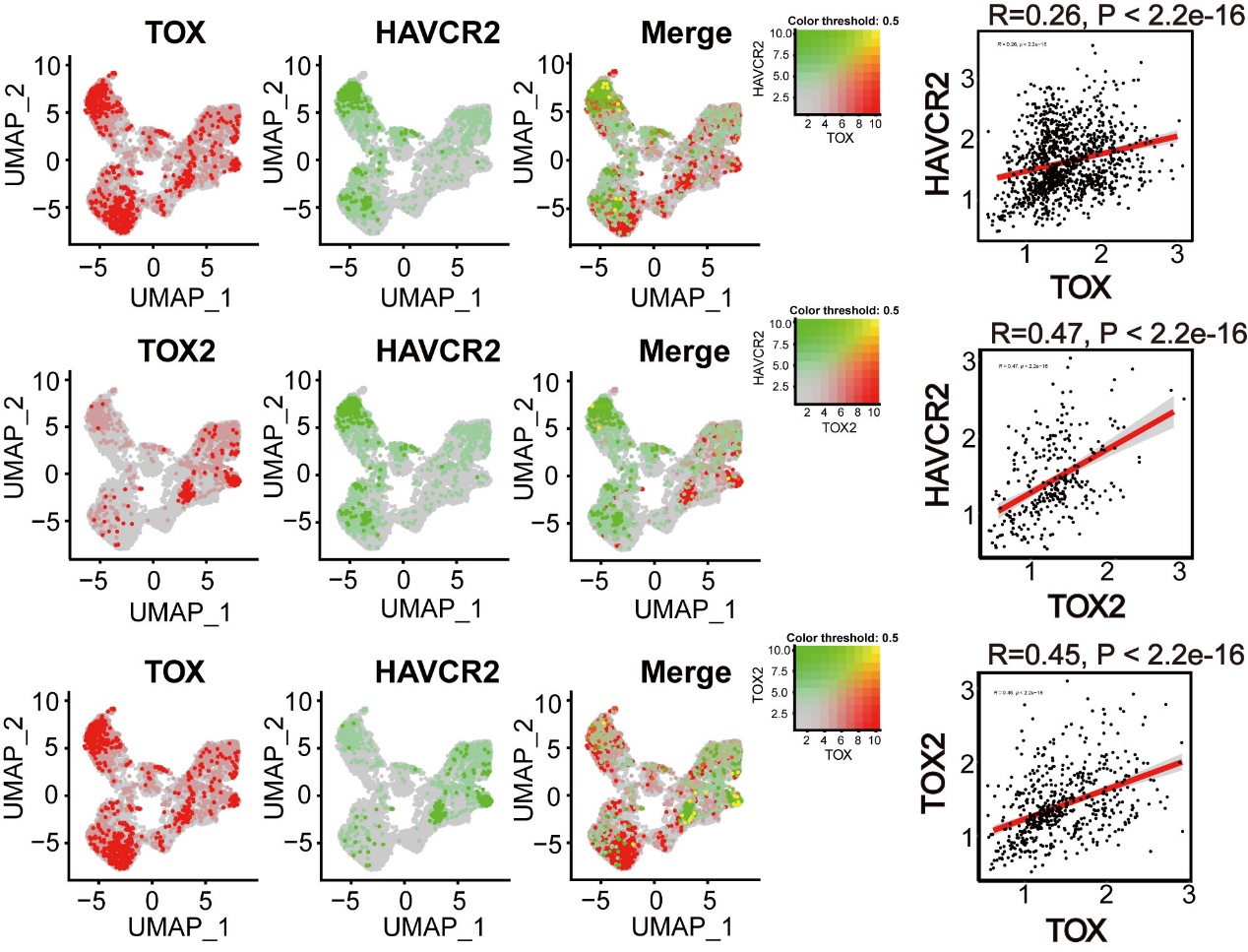


**Figure 1. Correlation of *TOX, TOX2,* and *HAVCR2* in tumor-infiltrating T cells (TILs).**

UMAP plots showing the co-expression of *TOX*, *TOX2,* and *HAVCR2* and correlation analysis between *TOX* and *TOX2*, *TOX* and *HAVCR2,* and *TOX2* and *HAVCR2* across individual single CD3+ TIL clusters from nasopharyngeal carcinoma patients based on scRNA-seq in GEO database (GSE: 162025). Red/green dots represent the expression level and distribution of *TOX*, *TOX2,* and *HAVCR2* in CD3+ TILs as indicated (first/second panels), and yellow dots indicate co-expression between *TOX*, *TOX2,* and *HAVCR2* in CD3+ TILs as indicated (third panel). The fourth panel shows the correlation between *TOX*, *TOX2,* and *HAVCR2* in CD3+ TILs as indicated.


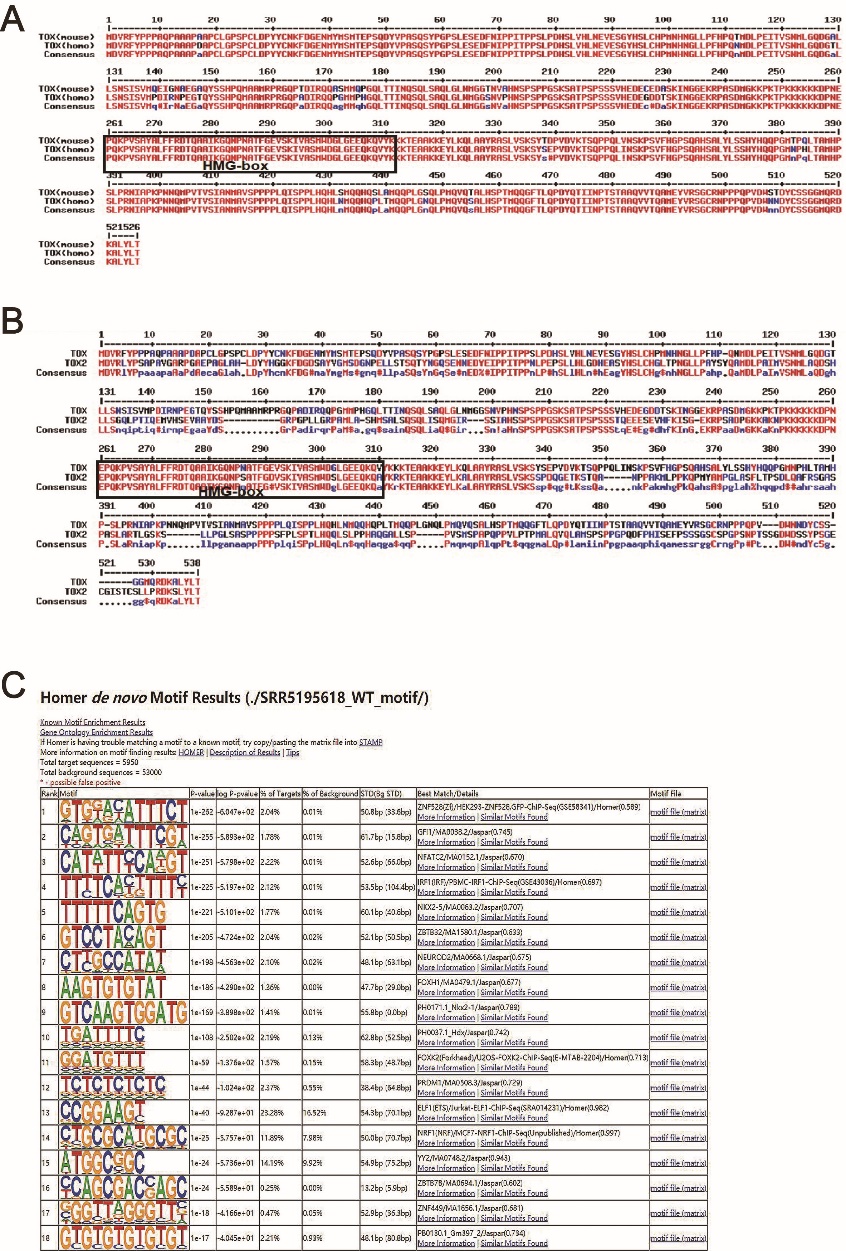


**Figure 2. Mouse TOX motif analysis and protein sequence alignment. (A)** Alignment analysis of mouse and human TOX protein sequences in the HMG-box domain. **(B)** Alignment analysis of the HMG-box domain of human TOX and TOX2 protein sequences. The blue amino acids indicate the different amino acid sequences of the two proteins. **(C)** Homer motif analysis identified 18 mouse TOX DNA-binding motifs.


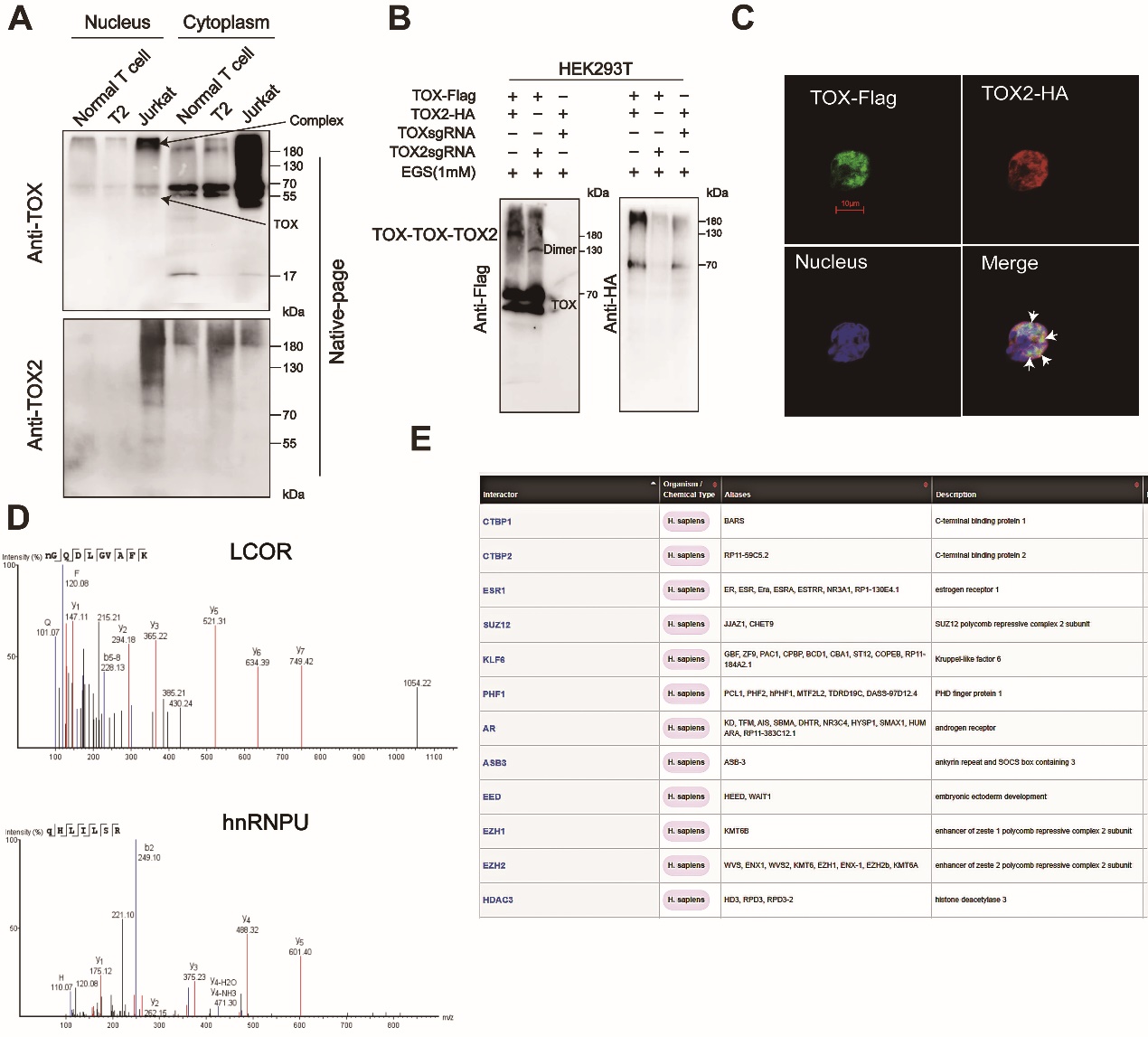


**Figure 3. Distribution and components of the TOX/TOX2 complex. (A)** Native-page electrophoresis and immunoblotting using anti-TOX or anti-TOX2 antibodies showing the molecular weight of the TOX-TOX2 complex, TOX-TOX dimer, and TOX monomer in the nucleus or cytoplasm of OKT3-stimulated normal T cells, T2, and Jurkat cells, as indicated. **(B)** Chemical cross-linking and immunoblotting analysis using anti-Flag or anti-HA antibodies showing the molecular weight of the TOX/TOX2 complex, TOX/TOX dimer, and TOX monomer in HEK293T cells transfected with pCDH plasmid vector containing TOX-Flag or TOX2-HA, and CRISPR-Cas9 plasmid vector containing TOXsgRNA or TOX2sgRNA as indicated for 48 h. Intracellular chemical cross-linking was performed by the transmembrane reagent EGS (1 mM). **(C)** Immunofluorescence analysis showing the cellular localization of TOX-Flag and TOX2-HA in HEK293T cells transfected with pCDH plasmid vector containing TOX-Flag (green) and TOX2-HA (red). Cell nuclei were stained with DAPI (blue). Yellow: co-localization of TOX-Flag and TOX2-HA. TOX was incubated with anti-Flag and TOX2 with anti-HA. **(D)** Mass spectrometric analysis of peptides from LCOR and hnRNPU proteins in co-IP enrichment pull down of anti-TOX in nuclear proteins from OKT3-stimulated T cells overexpressing TOX2-NLS-HA. **(E)** Biogrid database analysis showing proteins that interact with LCOR.


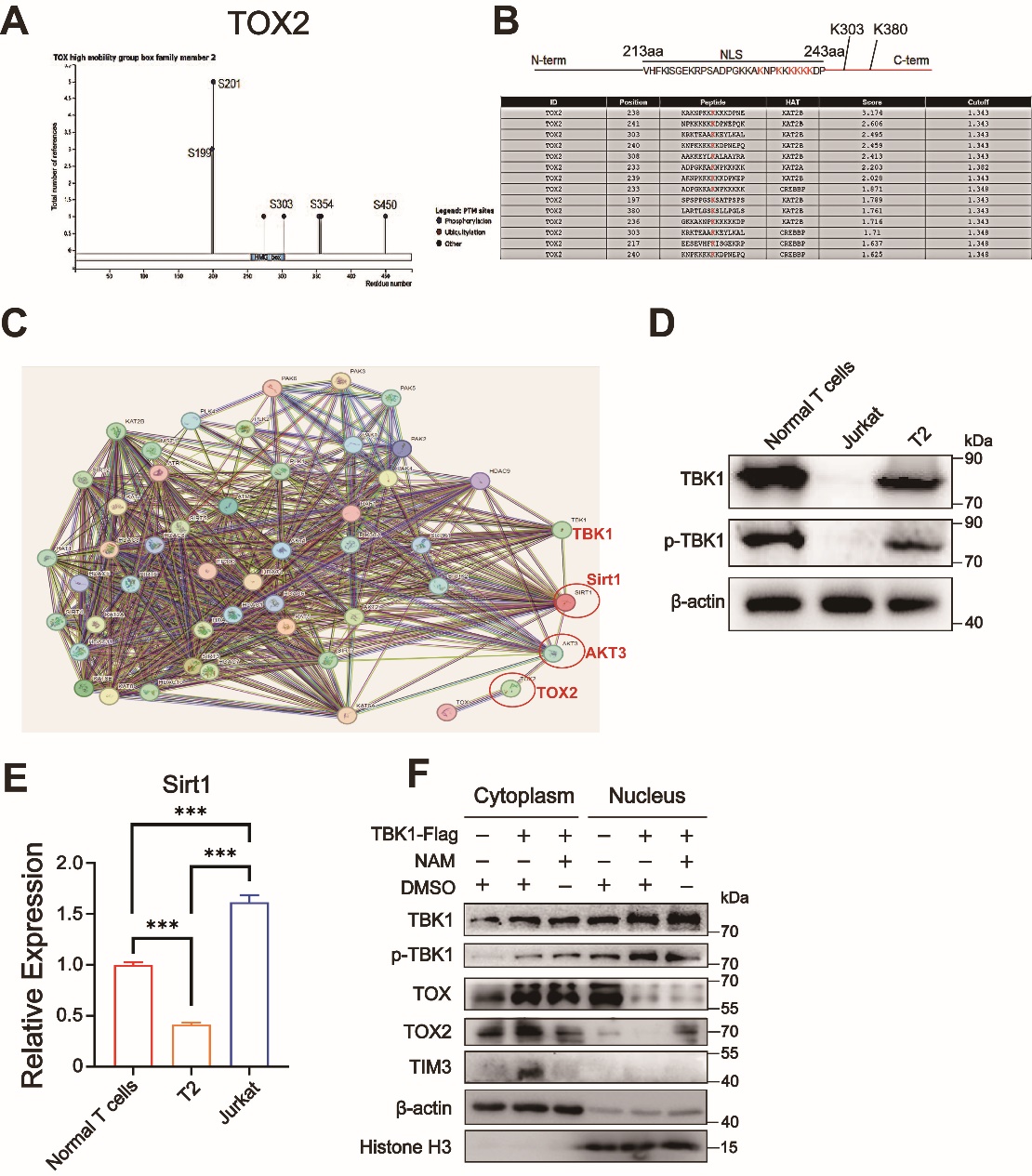


**Figure 4. *In silicon* and experimental analysis of the regulation of TOX2 nuclear translocation. (A)** TOX2 protein phosphorylation sites were predicted using the Uniprot database (https://www.uniprot.org/). S199/201/303/354/450 indicated the predicted serine phosphorylation sites in the TOX2 protein. **(B)** Lysine (K) acetylation sites in TOX2 were predicted by the GPS-PAIL database (http://pail.biocuckoo.org/). **(C)** The network between TOX2, protein kinases, acetyltransferases, and deacetylases were predicted using the STRING database. **(D)** Immunoblotting revealed the expression of TBK1 and p-TBK1 in normal T cells, T2, and Jurkat cells. β-actin was used as a loading control. **(E)** qPCR of the relative expression of Sirt1 in OKT3-stimulated normal T cells, T2, and Jurkat cells. **(F)** Immunoblot analysis of TBK1, p-TBK1, TOX, TOX2, and TIM3 levels in the nucleus and cytoplasm of TBK1-Flag-Jurkat cells with or without NAM treatment for 24 hours. β-actin and histone H3 were included as controls. Data are means ± SEM. **P < 0.01, ***P < 0.001, one-way ANOVA (E). Images are representative of three independent experiments.


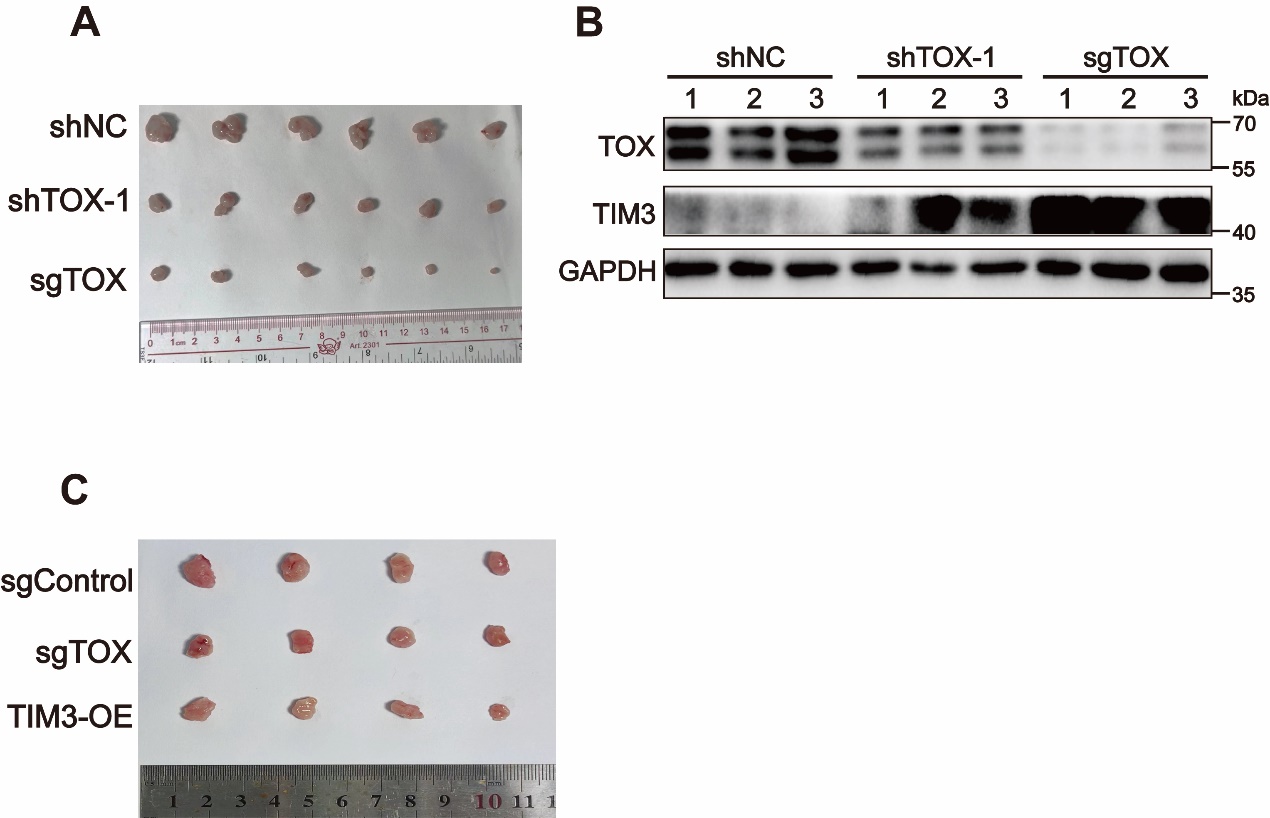


**Figure 5. Analysis of subcutaneous tumor tissue from nude mice. (A)** Xenograft tumors from shNC-, shTOX-1-, and sgTOX-Jurkat tumors in nude mice (n = 6 per group). (**B**) Immunoblot of the expression of TOX and TIM3 in tumor tissues from shNC-, shTOX-1-, and sgTOX-Jurkat tumors. GAPDH was used as a loading control. (C) Xenograft tumors from sgControl-, sgTOX-, and TIM3-OE-Jurkat tumors in nude mice (n = 4 per group).
